# Genome-wide identification and expression analysis of Dmrt genes in bivalves

**DOI:** 10.1186/s12864-023-09536-6

**Published:** 2023-08-15

**Authors:** Quanchao Wang, Tiangui Cao, Chunde Wang

**Affiliations:** 1 Yantai Institute of Coastal Zone Research, Chinese Academy of Sciences, Yantai, 264003 China; 2 Key Laboratory of Ecological Warning, Protection & Restoration for Bohai Sea, Ministry of Natural Resources, Qingdao, 266061 China

**Keywords:** Bivalve, Dmrt, Phylogeny, Gene expression

## Abstract

In recent years, some common themes in the development of sex-specific traits in different animal lineages have started to emerge since the discovery of the Dmrt (doublesex-mab3-related transcription factor gene) genes. Bivalves are characterized by a diversity of sexual systems, including simultaneous hermaphroditism, sequential hermaphroditism, and strict gonochorism. However, to date, no research has focused on the genome-wide characterization and analysis of Dmrt genes in bivalves. In this study, the identification and analysis of Dmrt genes in 15 bivalves were performed using bioinformatics methods. A total of 55 Dmrt genes were retrieved in the studied bivalve genomes. The number of Dmrt genes in different species ranged from 3 to 5. The phylogenetic tree showed that Dmrt genes in bivalves can be subdivided into 5 classes: the Dmrt2-like class, Dmrt3-like class, Dmrt4/5-like class, Dsx-like class, and scallop-specific Dmrt class. The Ka/Ks ratios suggested that all Dmrt classes underwent purifying selection pressure. Furthermore, the spatiotemporal expression of Dmrt genes in four bivalve species suggested that different Dmrt genes may have different functions, and scallop-specific Dmrt genes may play a key role in sex determination/differentiation. In general, this study provides a molecular basis for in-depth examination of the functions of Dmrt genes and phylogenomic analyses in bivalves.

**Supplementary Information:**

The online version contains supplementary material available at 10.1186/s12864-023-09536-6.

## Introduction

The founding member of the Dmrt (double sex/male-abnormal-3 related transcription factor) genes was first formally identified in *Drosophila*, where the Dsx gene controls somatic sexual differentiation via alternatively spliced, male- and female-specific isoforms (dsxM and dsxF, respectively) [1]. Subsequently, different Dmrt members have been identified and proven to participate in the control of sex determination/differentiation in other organisms. For example, the Z-linked gene Dmrt1 is vital for male sex determination in chickens [1], and a W-linked Dmrt gene (DM-W) participates in primary ovary development in *Xenopus laevis* [2]. A similar phenomenon was also observed in aquatic animals. In the medaka *Oryzias latipes*, a Y-specific DMY gene, as a copy of autosome Dmrt1, was found to be the master sex-determining gene inducing male formation. In general, given the key role of the Dmrt gene in sex determination/differentiation, Dmrt genes have been intensively investigated [3–5].

The members of the Dmrt gene family in different organisms showed substantial differences. For instance, 8 Dmrt members have been found in several mammals [6, 7], and 7 members of the Dmrt gene family have been identified in many teleosts [8]. Only 4 Dmrt members have been identified in *Drosophila melanogaster* [9]. Little is known about Dmrt genes in aquatic invertebrates, and no comprehensive survey and analysis of Dmrt genes has been conducted in bivalves. Bivalves, including clams, oysters, mussels, and scallops, were characterized by a shell that was divided from front to rear into left and right valves. A diversity of sexual systems has been found in bivalves, including simultaneous hermaphroditism, sequential hermaphroditism, and strict gonochorism [10]. For example, *Crassostrea virginica, Mizuhopecten yessoensis* and *Chlamys farreri* are gonochoristic, while *Argopecten purpuratus* and *Pecten maximus* are hermaphroditic species. However, the role of Dmart genes in these quite diversified bivalves is not clear.

Previous studies have demonstrated that the Dmrt gene is associated with sex determination/differentiation in bivalves. For example, Dmrt is significantly differentially expressed between the ovaries and testes of scallops [11, 12], and RNAi of Dmrt can lead to the failure of gonadal differentiation in oysters [13]. However, many questions about the Dmrt gene in bivalves remain to be answered. For instance, how many types of Dmrt genes are present in bivalves? What are the potential functions of different types of Dmrt genes? With the genome decoding of many bivalves, it is now feasible to investigate genome-wide Dmrt genes. In the present study, genome-wide and comprehensive analyses of Dmrt genes were conducted. The findings from this study will lay a foundation for understanding the genomic organization and functional structure of the Dmrt genes in the available genomes of bivalves, which will be useful in the characterization of functional genomics.

## Materials and methods

### Identification of Dmrt sequences in bivalves

The Dmrt sequences in 15 bivalves were extracted using BLAST and HMM methods. First, the genomic sequences and annotation files of 15 species were downloaded from different databases (Supplementary Table S1). Second, BLAST (V2.11.0) [14] and HMMER (V3.2.1) [15] were used to search Dmrt sequences in each genome with the DM domain query (accession: PF00751) downloaded from Pfam (http://pfam.xfam.org/). The initial threshold expectation values for both the BLAST and HMMER searches were set to 1 × 10^− 5^ and 1.0, respectively. Third, all the candidate genes obtained by using BLAST and HMMER searches were merged, and redundant genes were removed. Finally, the nonredundant genes were checked for the presence of the DM domain according to an E-value (10^− 5^) by online SMART analysis [16]. When two or more transcripts were annotated for a gene from alternative splicing, the longest form with a DM domain was selected. The length of the amino acid sequence (AA), molecular weight (MW), isoelectric point (pI) and total average hydrophilicity (GRAVY) of Dmrt proteins were predicted using TBtools software (version 1.098) [17].

### Phylogenetic analyses of the Dmrt gene family

A set of Dmrt protein sequences in different species was first obtained from the NCBI, JGI and UniProt databases (Supplementary Table S2). All retrieved Dmrt proteins and those identified in bivalves were used to perform phylogenetic analysis. Multiple sequence alignments of all Dmrt proteins were first generated using MAFFT v7.158b [18] with default parameters. Then, the phylogenetic tree was constructed using IQ-TREE v2.2.0 [19] with the option: -m MFP --bnni -B 1000 -T AUTO. The phylogenetic tree was visualized using iTOL (interactive tree of life) software [20] with the following settings: midpoint root and nonsorting leaf.

### Sequence analyses and genomic distribution of Dmrts

The Batch SMART plug-in in TBtools software (version 1.098) [17] was used to identify the conserved domains of Dmrt genes, and the iTOL (interactive tree of life) online tool was used for visualization [20]. The general feature format (GFF3) file was used to retrieve the Dmrt gene structure and exon information. The conserved motifs of the Dmrt gene family were predicted using MEME [21] with the following parameters: maximum length of the conserved motif, 50; minimum length, 6, largest number, 20, and default values for other parameters. Conserved motifs and gene structure were visualized using TBtools software (version 1.098) [17].

### Nonsynonymous (Ka) to synonymous substitution (Ks) ratio (Ka/ks) analysis

To understand the evolutionary rates of the Dmrt genes among scallops, a Bayesian inference approach for site-specific positive selection and purifying selection was used to estimate two types of substitution events by calculating the ratio of nonsynonymous (Ka) to synonymous substitutions (Ks) by the Selecton Server [22] with four evolutionary models (M5, M7, M8, and M8a).

### Expression profiling of Dmrt in three bivalves

To understand the expression patterns of Dmrt genes in bivalves, publicly available RNA-seq data from *A purpuratus*, *M. yessoensis, Mytilus coruscus*, and *Mercenaria mercenaria* were downloaded from the NCBI SRA database (Supplementary Table S3). Raw RNA sequencing reads were trimmed using the NGStoolkit program [23] with the default parameters. Then, the reference genome was indexed, and the clean reads were mapped to the reference genome using HISAT2 [24]. After the resulting SAM files were converted into BAM files and sorted using SAMtools [25], the FPKM value of each gene was determined using StringTie v2.1.7 [26] based on the annotated gff file. Heatmaps of the gene expression levels were generated using the ggplot2 package in R software [27].

## Results

### Identification and characterization of Dmrt in bivalves

A total of 55 Dmrt genes were found in 15 bivalve genomes. The number of Dmrt genes in bivalve genomes ranged from 3 to 5. The amino acid sequences of all identified Dmrt genes are listed in Supplementary Table S4. The characteristics of all the identified proteins in 15 bivalves, including coding sequence length, number of amino acids, molecular weight, theoretical pI, instability index, aliphatic index, and grand average of hydropathicity (GRAVY), were predicted and are listed in Table 1. The results showed that the biophysical properties of different Dmrt proteins were relatively stable. AA length ranged from 164 to 3551, with a mean of 452.81. The molecular weight ranged from 19638.64 to 396231.09 Da, and the PI values ranged from 4.94 to 10.04. Additionally, all the Dmrt members have an instability index greater than 40.

**Table 1:** Protein sequence features of identified Dmrts in bivalves

Species	Protein ID	Gene ID	AA	MW	PI	INS	AIN	GRAVY
*Argopecten irradians irradians*	evm.model.Contig349.42	Aii|evm.model.Contig349.42	368	40037.12	8.69	71.08	58.18	-0.63
	evm.model.Contig349.40	Aii|evm.model.Contig349.40	385	42805.32	7.97	60.07	59.06	-0.65
	evm.model.Contig6.279	Aii|evm.model.Contig6.279	305	33667.14	8.39	63.00	60.13	-0.57
*Argopecten purpuratus*	evm.model.scaffold_95.77	Ap|evm.model.scaffold_95.77	368	40010.01	8.69	71.44	57.12	-0.65
	evm.model.scaffold_95.76	Ap|evm.model.scaffold_95.76	385	42829.34	7.57	61.30	59.32	-0.65
	evm.model.scaffold_235.403	Ap|evm.model.scaffold_235.403	301	33183.54	8.39	67.21	58.67	-0.60
*Chlamys farreri*	CF57815.1	Cf|CF57815.1	385	42677.04	7.58	58.06	63.90	-0.63
	CF58131.2	Cf|CF58131.2	369	40004.09	8.66	68.46	59.08	-0.60
	CF3811.22	Cf|CF3811.22	354	40486.72	9.67	48.98	78.42	-0.57
	CF42417.14	Cf|CF42417.14	364	40429.24	8.95	67.07	73.13	-0.51
*Crassostrea gigas*	NP_001295834.1	Cg|NP_001295834.1	359	39077.98	8.71	64.65	57.38	-0.63
	XP_011427033.2	Cg|XP_011427033.2	390	42775.04	6.89	48.39	60.82	-0.65
	XP_011441049.2	Cg|XP_011441049.2	303	33730.61	9.42	52.93	63.43	-0.51
*Crassostrea virginica*	XP_022319926.1	Cv|XP_022319926.1	361	39081.06	8.67	70.80	59.78	-0.58
	XP_022317913.1	Cv|XP_022317913.1	394	43071.39	7.62	49.76	67.36	-0.57
	XP_022333988.1	Cv|XP_022333988.1	358	40046.83	9.41	55.41	50.14	-0.73
*Cyclina sinensis*	evm.model.Hic_asm_11.338	Cs|evm.model.Hic_asm_11.338	388	42625.61	8.59	56.58	57.14	-0.74
	evm.model.Hic_asm_11.400	Cs|evm.model.Hic_asm_11.400	388	42625.61	8.59	56.58	57.14	-0.74
	evm.model.Hic_asm_11.1174	Cs|evm.model.Hic_asm_11.1174	378	41376.62	8.05	44.63	69.50	-0.57
*Dreissena polymorpha*	KAH3782801.1	Dp|KAH3782801.1	370	40723.62	8.53	52.20	60.97	-0.55
	KAH3696108.1	Dp|KAH3696108.1	435	47961.74	8.40	57.30	64.16	-0.65
	KAH3699546.1	Dp|KAH3699546.1	329	36383.80	9.16	52.08	62.89	-0.68
	KAH3721156.1	Dp|KAH3721156.1	164	19638.64	9.16	51.03	64.27	-0.56
*Mercenaria mercenaria*	XP_045157593.1	Mme|XP_045157593.1	389	42923.17	8.55	56.88	59.51	-0.68
	XP_045159713.1	Mme|XP_045159713.1	367	40402.13	7.65	63.66	61.12	-0.59
	XP_045156965.1	Mme|XP_045156965.1	355	40092.04	10.04	50.80	72.48	-0.69
	XP_045157038.1	Mme|XP_045157038.1	378	41407.62	8.24	45.37	67.96	-0.57
	XP_045157053.1	Mme|XP_045157053.1	300	33105.37	8.54	51.99	69.67	-0.58
*Mizuhopecten yessoensis*	XP_021377274.1	My|XP_021377274.1	369	39967.03	8.66	70.40	58.81	-0.60
	XP_021368788.1	My|XP_021368788.1	354	40273.31	9.60	51.66	75.14	-0.61
	XP_021377273.1	My|XP_021377273.1	385	42857.24	6.83	57.01	62.36	-0.67
	XP_021353714.1	My|XP_021353714.1	306	34274.91	8.24	64.64	60.56	-0.60
*Mytilus coruscus*	CAC5398878.1	Mc|CAC5398878.1	349	38203.12	8.88	64.37	59.05	-0.61
	CAC5404148.1	Mc|CAC5404148.1	333	38283.01	9.69	56.76	82.52	-0.57
	CAC5360634.1	Mc|CAC5360634.1	363	40385.17	8.80	59.56	54.60	-0.75
	CAC5397186.1	Mc|CAC5397186.1	248	28177.66	9.01	56.27	54.96	-0.89
*Mytilus edulis*	CAG2209978.1	Me|CAG2209978.1	349	38104.97	8.88	68.62	58.80	-0.60
	CAG2252366.1	Me|CAG2252366.1	318	36776.24	9.79	66.18	81.79	-0.62
	CAG2226664.1	Me|CAG2226664.1	364	40444.29	8.80	63.13	56.07	-0.74
	CAG2232556.1	Me|CAG2232556.1	189	21260.00	9.20	51.21	52.54	-0.87
*Mytilus galloprovincialis*	VDI24477.1	Mg|VDI24477.1	349	38133.03	8.88	68.62	59.34	-0.59
	VDI42071.1	Mg|VDI42071.1	327	37709.39	9.86	61.57	83.73	-0.59
	VDI32052.1	Mg|VDI32052.1	3551	396231.09	4.94	35.44	81.39	-0.25
	VDI03798.1	Mg|VDI03798.1	249	28236.86	9.12	55.97	56.31	-0.85
*Ostrea edulis*	XP_048763391.1	Oe|XP_048763391.1	358	39016.87	8.54	66.16	58.63	-0.60
	XP_048761857.1	Oe|XP_048761857.1	391	43125.39	6.95	44.13	62.66	-0.64
	XP_048736153.1	Oe|XP_048736153.1	262	29928.35	9.54	70.86	61.03	-0.71
	XP_048736174.1	Oe|XP_048736174.1	262	29928.35	9.54	70.86	61.03	-0.71
*Pecten maximus*	XP_033737544.1	Pm|XP_033737544.1	369	40256.13	8.57	69.66	55.61	-0.70
	XP_033738864.1	Pm|XP_033738864.1	351	39626.69	9.70	51.09	81.82	-0.51
	XP_033737545.1	Pm|XP_033737545.1	385	42746.16	7.59	59.49	60.36	-0.64
	XP_033733655.1	Pm|XP_033733655.1	303	33533.10	8.27	64.84	62.81	-0.56
*Saccostrea glomerata*	Sgl006992-mRNA1	Sg|Sgl006992-mRNA1	357	38971.85	8.66	67.68	58.24	-0.64
	Sgl014397-mRNA1	Sg|Sgl014397-mRNA1	391	43166.57	8.31	42.83	63.12	-0.65
	Sgl011295-mRNA1	Sg|Sgl011295-mRNA1	273	30861.32	9.68	59.16	56.45	-0.70

AA, amino acid length; MW, molecular weight, KD; PI, isoelectric point; INS, instability index; AIN, aliphatic index; GRAVY, grand average of hydropathy

### Phylogenetic analysis of Dmrt genes

**Fig. 1:**
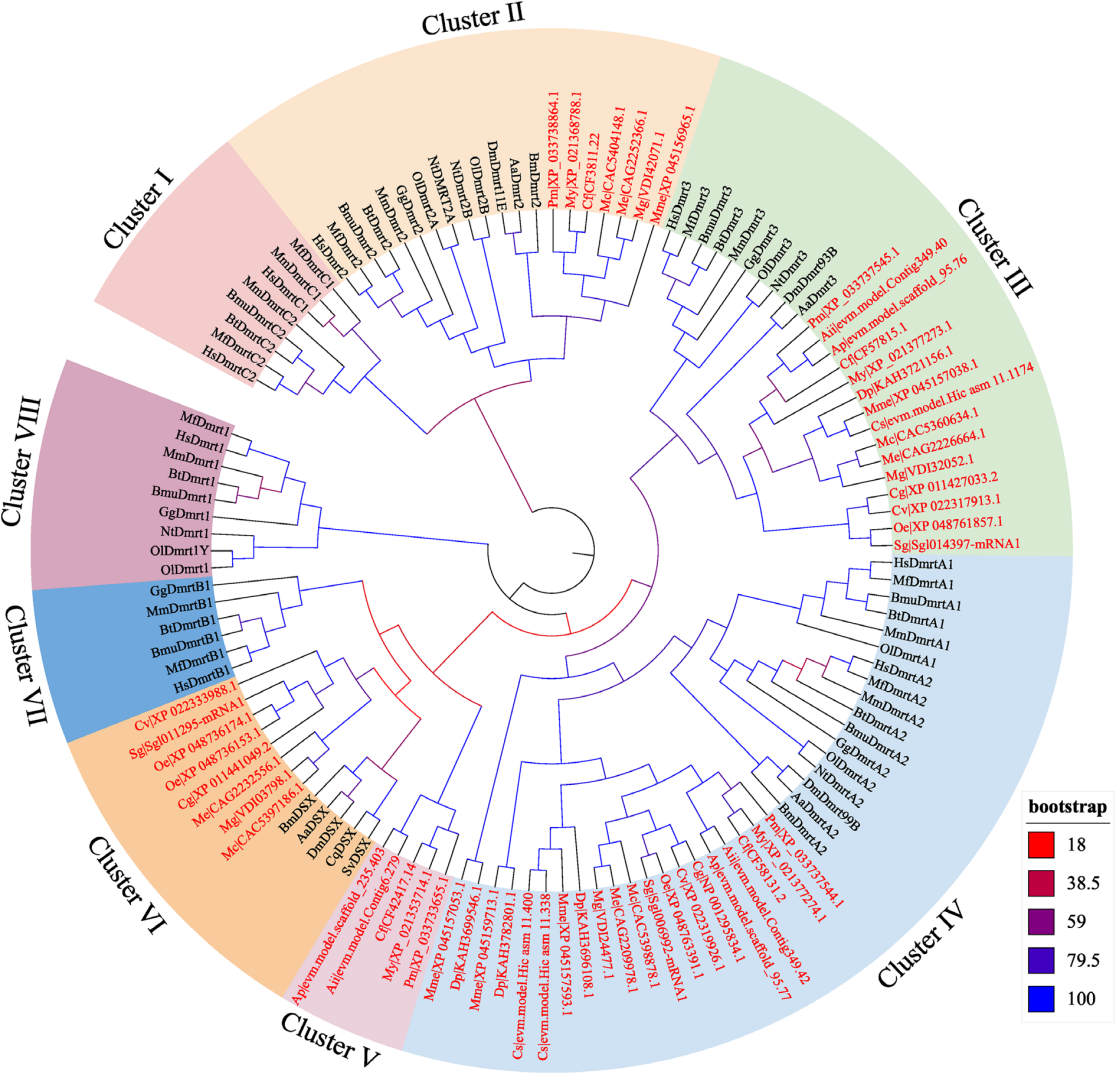
Phylogenetic tree of Dmrt protein sequences

To explore the evolutionary history of Dmrt genes in bivalves, a phylogenetic analysis was performed using the DM-domain protein sequences from vertebrates and invertebrates. As shown in Fig. 1, the Dmrt proteins were clustered into 8 branches (Cluster I to Cluster VII), but bivalve DM-domain proteins were grouped into only five clusters, Cluster II, III, IV, V, and VI. The Cluster II, corresponding to Dmrt2, contains 7 bivalve DM-domain proteins. Cluster III corresponds to a Dmrt3 class and contains 15 bivalve Dmrt proteins. Twenty bivalve Dmrt proteins were grouped into the Dmrt4/5 class (Cluster IV). Interestingly, 5 scallop Dmrt proteins evolved into a separate cluster (Cluster V). In addition, 8 Dmrt proteins from 7 bivalves shared a close phylogenetic relationship with invertebrate Dsx genes.

### Conserved domain, gene structure and motif analysis of Dmrt

**Fig. 2:**
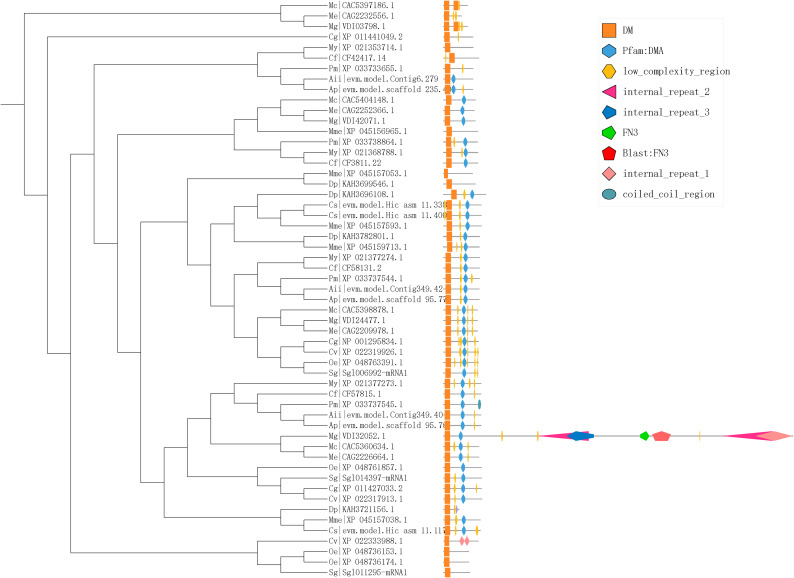
Conserved domain structures of identified Dmrt genes in bivalves

**Fig. 3:**
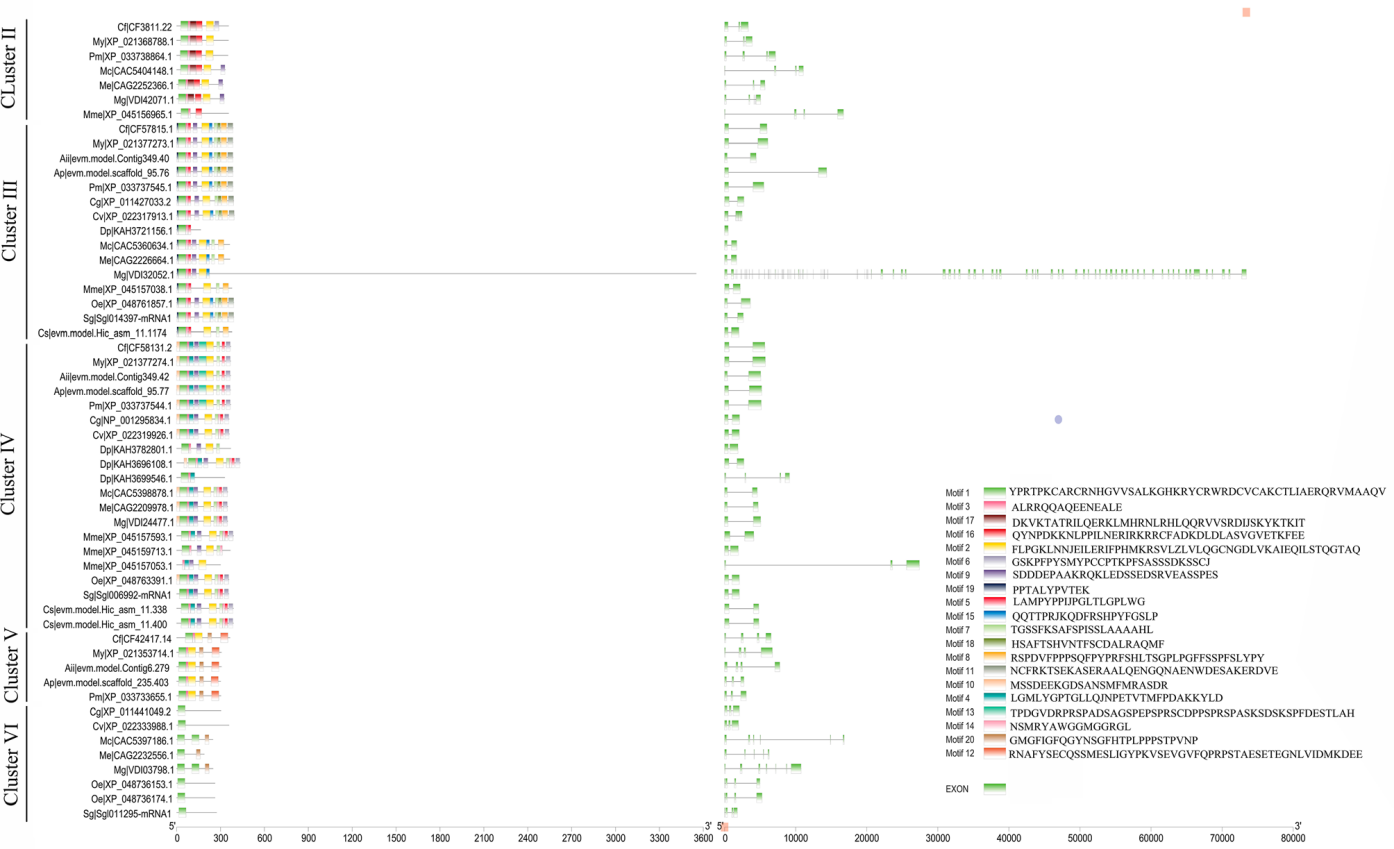
Motif composition and exon‒intron structures of bivalve Dmrt genes

A batch SMART search showed that all Dmrt genes included one to two DM domains, and some Dmrt genes contained Pfam:DMA and low_complexity_region (Fig. 2). Exon diversification among Dmrt genes is displayed in Fig. 3. Exon numbers of Dmrt genes in 15 bivalves ranged from 1 to 90. The exon numbers of Dmrt genes in Cluster II varied from 2 to 4. The majority of members in Clusters III and IV have two exons. In Cluster V, 4 Dmrt genes had three exons, and one Dmrt gene had 4 exons. All members in Cluster VI have no fewer than 4 exons. In addition, although all the predicted Dmrt proteins contain motif 1, the proteins in the same cluster also have similar motif structural features. For example, all members in Cluster V have the same motif structure and are quite different from the other clusters.

### Selective pressure analysis

The Ka/Ks ratios of orthologous Dmrt genes among different clusters are shown in Table 2. The results indicated that the Ka/Ks ratios of the sequences from different Dmrt groups were significantly different. The highest Ka/Ks values in Cluster VI indicated a higher evolutionary rate within members of the Cluster IV. In contrast, the relatively low Ka/Ks values in Clusters IV and V implied a lower evolutionary rate or selective constraint within these groups. However, despite the differences in Ka/Ks values, all the estimated Ka/Ks values were substantially lower than 1, suggesting that the Dmrt sequences within each cluster are under purifying selection pressure.

**Table 2:** Selection analysis of the different Dmrt classes in bivalves

Cluster	Selection model	Ka/Ks*	Log-likehood	Numbers of positive selection site
Cluster II	M8 (beta + w > = 1)	0.32	-5508.06	0
	M8a (beta + w = 1)	0.33	-5509.29	0
	M7 (beta)	0.35	-5509.89	0
	M5 (gamma)	0.34	-5511.88	0
Cluster III	M8 (beta + w > = 1)	0.23	-23707.4	0
	M8a (beta + w = 1)	0.23	-23701.6	0
	M7 (beta)	0.22	-23696.5	0
	M5 (gamma)	0.24	-23730.9	0
Cluster IV	M8 (beta + w > = 1)	0.16	-14240.5	0
	M8a (beta + w = 1)	0.17	-14237.8	0
	M7 (beta)	0.16	-14226.8	0
	M5 (gamma)	0.17	-14259.7	0
Cluster V	M8 (beta + w > = 1)	0.17	-3054.44	0
	M8a (beta + w = 1)	0.17	-3054.53	0
	M7 (beta)	0.18	-3056.04	0
	M5 (gamma)	0.18	-3055.55	0
Cluster VI	M8 (beta + w > = 1)	0.51	-5480.61	0
	M8a (beta + w = 1)	0.48	-5479.98	0
	M7 (beta)	0.52	-5482.33	0
	M5 (gamma)	0.59	-5493.41	18

### Spatiotemporal expression profile in bivalves

**Fig. 4:**
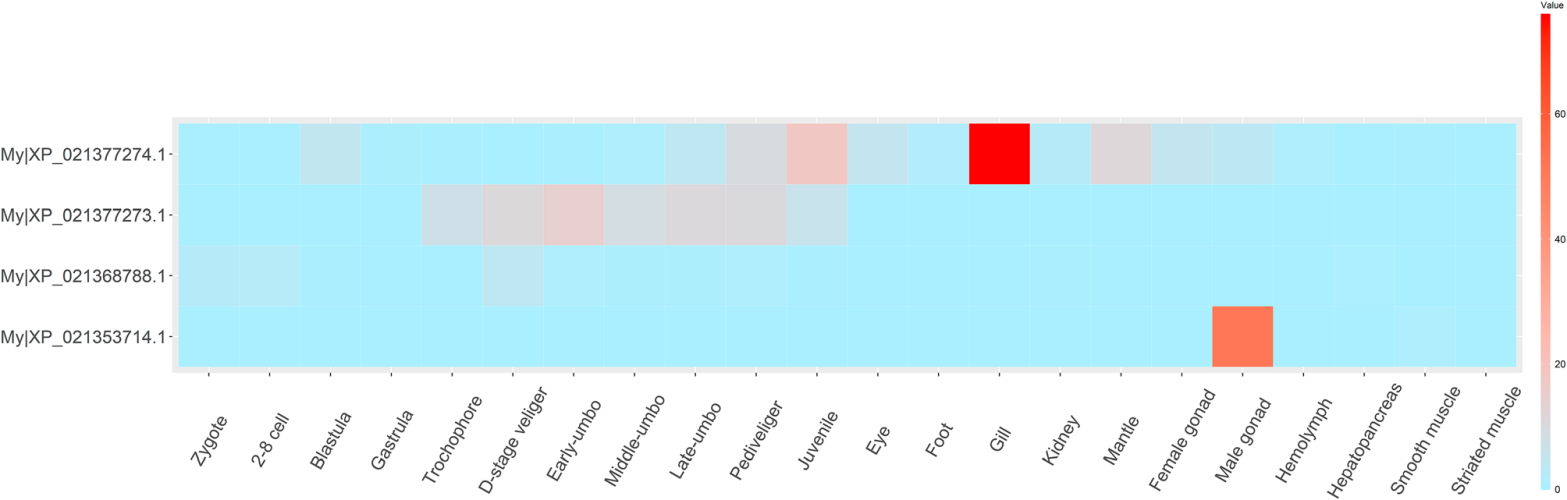
Dmrt gene expression patterns at different developmental stages and in different adult tissues of *Mizuhopecten yessoensis*

**Fig. 5:**
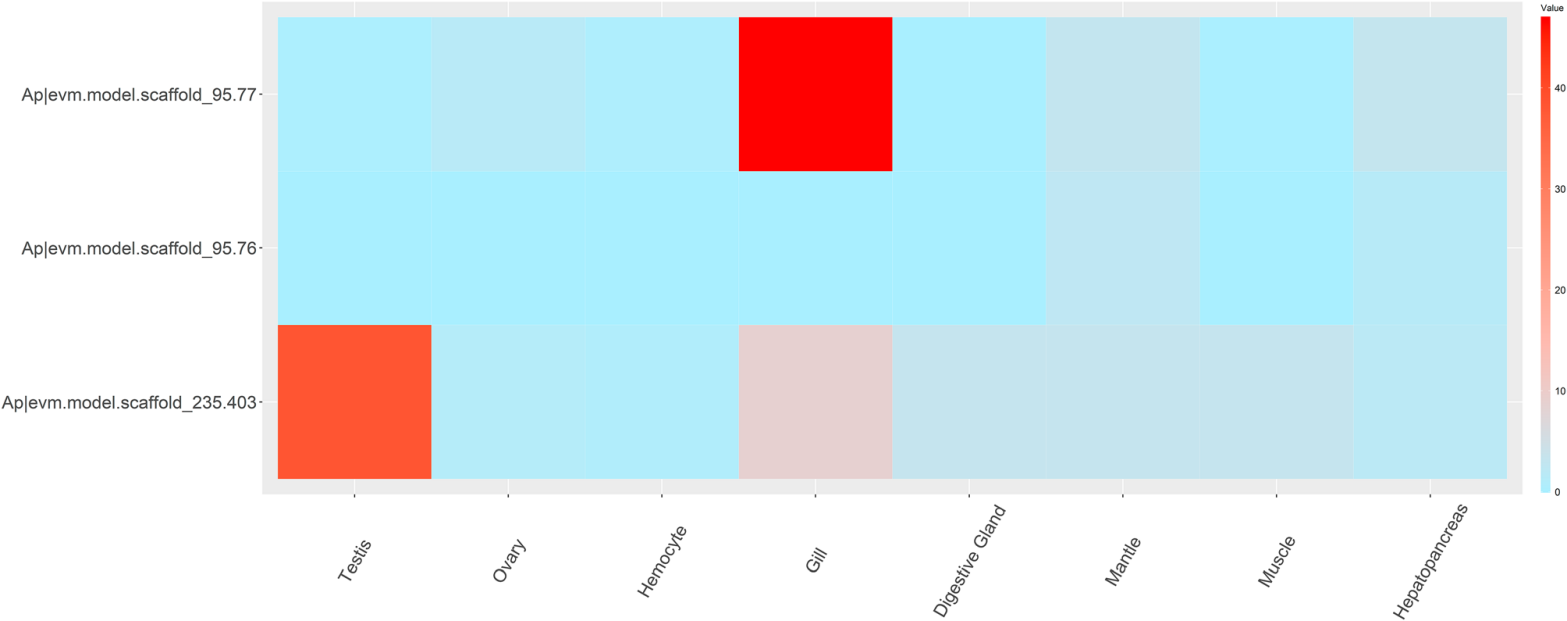
Dmrt gene expression patterns in different adult tissues of *Argopecten purpuratus*

RNA-seq datasets from different tissues and developmental stages of Dmrt genes in *M. yessoensis* were analyzed to investigate the expression patterns of different Dmrt genes. As shown in Fig. 4, the My|XP_021353714.1 and My|XP_021368788.1 in most developmental stages were not expressed (FPKM = 0) or expressed at low levels (FPKM < 1). From the trochophore stage, the expression of My|XP_021377273.1 showed a tendency to increase first followed by a decrease, while My|XP_021377274.1 exhibited high expression levels in juvenile stages. At the adult stage, the My|XP_021368788.1 and My|XP_021377273.1 were rarely expressed in all tissues, while the My|XP_021353714.1 and My|XP_021377274.1 showed specificity of high expression in male gonads and gills, respectively. Similar results can be learned in the hermaphrodite scallop *A*. *purpuratus* (Fig. 5). Ap|evm.model.scaffold_235.403 and Ap|evm.model.scaffold_95.77 were also specifically highly expressed in the testis and gill, respectively. The Ap|evm.model.scaffold_95.76 was rarely expressed in the studied tissues.

**Fig. 6:**
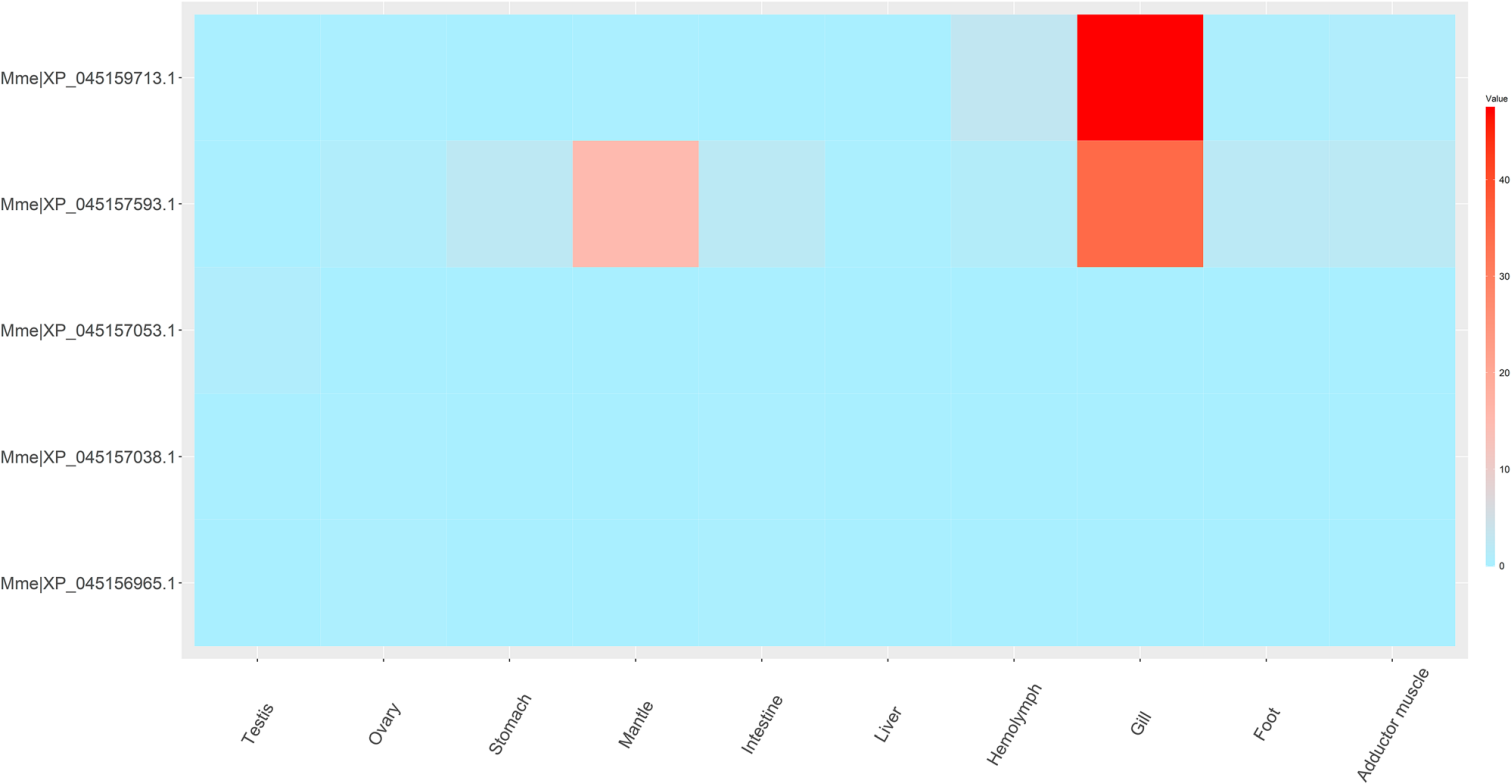
Dmrt gene expression patterns at different developmental stages and in different adult tissues of *Mytilus coruscus*

In *M. mercenaria* (Fig. 6), the Mme|XP_045157593.1 was expressed in multiple tissues, especially in gills. Similarly, the expression of Mme|XP_045159713.1 was also the highest in gills. However, the other Dmrt genes were not expressed (FPKM = 0) or were expressed at low levels (FPKM < 1) in all tissues.

**Fig. 7:**
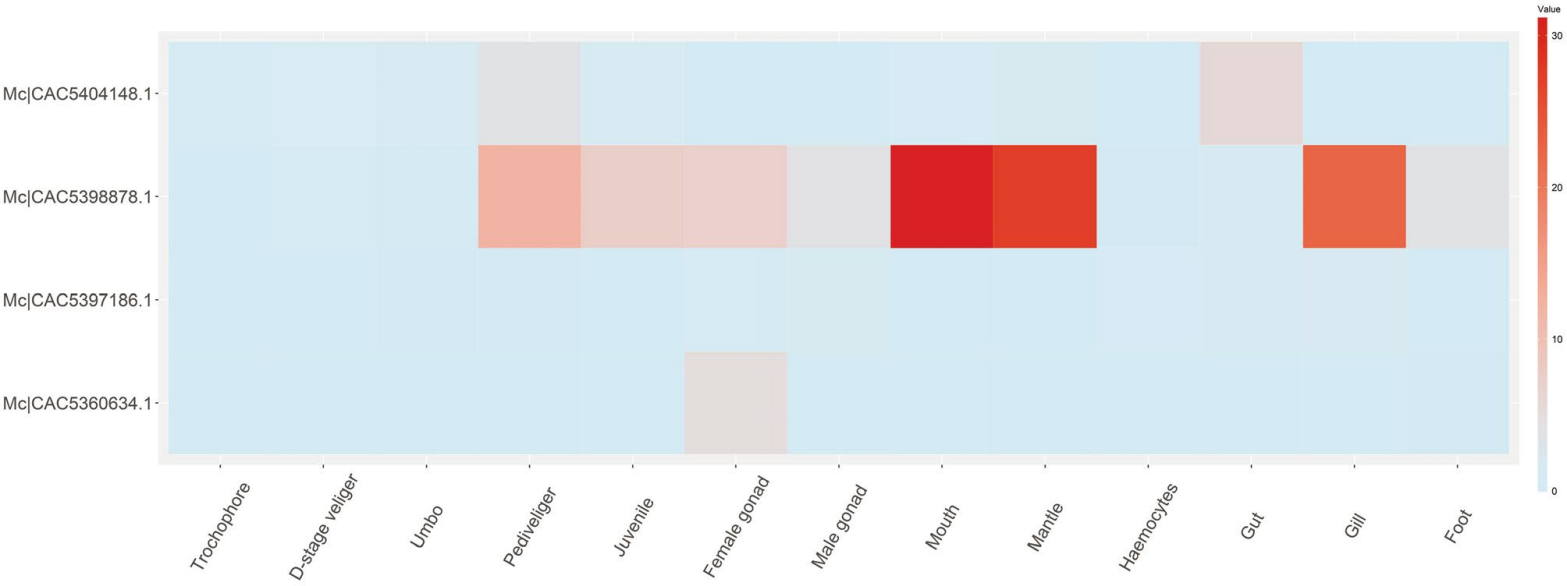
Dmrt gene expression patterns in different adult tissues of *Mercenaria mercenaria*

The spatiotemporal expression profile of Dmrt genes in *M. coruscus* showed that no Dmrt genes were expressed (FPKM = 0) or they were expressed at low levels (FPKM < 1) in the trochophores, D-stage veliger, and umbo larvae (Fig. 7). In the pediveliger and juvenile stages, the Mc|CAC5360634.1 and Mc|CAC5397186.1 were still not expressed, while the Mc|CAC5398878.1 and Mc|CAC5404148.1 presented different levels of expression. At the adult stage, the Mc|CAC5398878.1, Mc|CAC5404148.1, and Mc|CAC5397186.1 were expressed in different tissues, while Mc|CAC5360634.1 was highly expressed in female gonads.

## Discussion

Dmrt family genes have been considered sex-related genes because of their functions in sex determination/differentiation, testicular development, and embryo development [28, 29]. To date, genome-wide identification of Dmrt genes has been carried out in various animal groups but not in aquatic invertebrates [30–32]. In particular, a comprehensive survey and analysis of the Dmrt genes has not been conducted in bivalves. In this study, genome-wide identification of Dmrt genes was performed in 15 bivalve genomes. Three to five Dmrt genes have been identified in bivalve genomes. The number of Dmrt genes in bivalves was close to that of other invertebrates but lower than that observed in many teleosts. The difference in the number of Dmrt genes may be related to genome size and genomic duplication events [32]. Furthermore, the presence of different Dmrt genes in the animals seemed to be species specific. For example, Dmrt1 has only been identified in vertebrates, and Dmrt 6/7/8 is only present in mammals. In contrast, our results in this study showed that the Dmrt2-like, Dmrt3-like, Dmrt4/5-like and Dsx-like genes, but not the Dmrt1-like gene or Dmrt6/7/8-like gene, were identified in bivalves. Similar results were also found in Panarthropoda [33] and echinoderms [34].

Among the Dmrt genes identified in bivalves, some were identified in all the species studied, such as Dmrt3-like and Dmrt4/5-like genes, while the Dmrt2-like gene was only present in seven bivalves. Considering that Dmrt2 can be identified in the deuterostome [30, 34, 35], the origin and evolution of Dmrt2-like genes in bivalves should be studied further. Consistent with previous findings [30, 34, 35], the current phylogenetic analysis showed that Dmrt4 and Dmrt5 were clustered into a major branch, implying that these two types of genes originated from the same ancestor of Dmrt. The Dmrt4/5-like gene was identified in all bivalves and was duplicated in *Mercenaria mercenaria*, *Cyclina sinensis*, and *Dreissena polymorpha*. In addition, a Dsx-like gene class was found in the phylogenetic tree, although backed by low bootstrap values. This result may be related to little sequence conservation outside of the DM domains. Moreover, in this study, we identified a novel Dmrt class with unique exons and motifs that was phylogenetically distant from the other Dmrt members in scallops. Some novel Dmrt genes have also previously been identified in other aquatic invertebrates [34, 36, 37], suggesting that Dmrt genes in aquatic invertebrates may be distinct from those in other animals.

In the current study, the Dmrt4/5-like genes, including Ap|evm.model.scaffold_95.77, My|XP_021377274.1, Mc|CAC5398878.1, Mme|XP_045157593.1, and Mme|XP_045157593.1, were expressed in multiple tissues, especially in the gills of the four bivalves. Similar results can be found in other aquatic species. For instance, Dmrt4 is expressed only in the gills of *Xiphophorus maculatus* [38], and Dmrt4-like genes are highly expressed in the gills and mantle of *Ruditapes philippinarum* [39]. Previous reports have shown that Dmrt4 and Dmrt5 may be involved in neurogenesis. For example, in *Xenopus*, Dmrt4 and Dmrt5 are important regulators of olfactory placode neurogenesis [40, 41]. In *Drosophila*, Dmrt99B, an ortholog of Dmrt4/5, is required for initiating temporal patterning in medulla neuroblasts. Thus, it will be interesting to investigate whether Dmrt4/5 orthologs also play conserved roles in the neurogenesis of bivalves.

Previous studies have shown that the Dmrt2 gene participates in multiple biological processes, such as somitogenesis [42], oogenesis [43], and chondrocyte differentiation [44]. In the current study, the My|XP_021368788.1 was expressed in zygotes, 2–8 cells, and D-stage veliger, and the Mc|CAC5404148.1 was expressed from the umbo to juvenile stage. These results suggested that the Dmrt2-like genes might be involved in the early development of bivalves. To date, data on Dmrt3 genes are very limited. This gene has been confirmed to play a pivotal role in gonadal sex determination in fish [45] and spinal circuit function in mice (Andersson et al., 2012). The orthologs of Dmrt3 in the three bivalves showed different expression profiles, implying that these genes may perform different functions. However, it is worth noting that the Dmrt3-like (Mc|CAC5360634.1) gene is specifically expressed in the female gonad of *M*. *coruscus*. This result suggested that the Dmrt3-like gene may be an important gene involved in the ovary-determining pathway in *M*. *coruscus*. Moreover, the Dmrt3-like gene (Mg|VDI32052.1) in *Mytilus galloprovincialis* presents a unique gene structure, and the analysis based on multiple SRA datasets (Supplementary Table S3) did not detect the expression of this gene. Therefore, whether this gene is a pseudogene should be further verified.

In this study, the Dsx-like gene showed low expression in all tested stages and adult tissues of *M. coruscus*. Thus, the function of this type of gene remains to be further investigated. Interestingly, My|XP_021353714.1 was specifically expressed in the male gonad, suggesting that it may be an important gene involved in sex determination/differentiation. This result can also be learned in previous studies. For example, Li et al. (2016) showed that PyDMRT was testis-biased, and Li et al. (2018) suggested that the My|XP_021353714.1 (previously known as Dmrt1L) is a yang gene for determining the timing of sex differentiation in *M. yessoensis* [46]. Similar results were also found in *Argopecten irradians* [11], in which Dmrt1L showed male-biased expression in the gonad. In particular, the current study showed that the gene (Ap|evm.model.scaffold_235.403) from the hermaphrodite scallop *A. purpuratus* is also expressed specifically in the testis. Therefore, it is possible that the genes in Cluster V play pivotal roles in testis determination in scallops. In general, this study provides a molecular basis for Dmrt genes in bivalves.

## Conclusions

In this study, Dmrt genes were identified and analyzed in 15 bivalves. A total of 55 Dmrt genes were identified, and the number of Dmrt genes in bivalves ranged from 3 to 5. The phylogenetic tree showed that all Dmrts from bivalves were classed into 5 clusters, corresponding to the Dmrt2-like class, Dmrt3-like class, Dmrt4/5-like class, Dsx-like class, and scallop-specific Dmrt class. Furthermore, the Ka/Ks ratios suggested that all the Dmrt clusters underwent purifying selection pressure. The spatiotemporal expression profile in bivalves suggested that different Dmrt genes may have different functions, and the scallop-specific Dmrt gene may play an important role in sex determination/differentiation. In general, this study provides a molecular basis for in-depth functional examination of Dmrt genes and phylogenomic analyses in bivalves.

### Electronic supplementary material

Below is the link to the electronic supplementary material.

Supplementary Material 1

## Data Availability

The datasets generated and/or analyzed during the current study are available in the NCBI repository [PRJNA259405, PRJNA428789, PRJEB35330, PRJNA578350, PRJNA533175, PRJNA638823, PRJNA785550, PRJEB58207, PRJNA740305, PRJEB35351, and PRJNA376014], [PERSISTENT WEB LINK OR ACCESSION NUMBER TO DATASETS], cfbase [http://mgb.ouc.edu.cn/cfbase/html/], GIGADB [http://www.gigadb.org/dataset/100419], dbSROG [http://soft.bioinfo-minzhao.org/srog/#], and DRYAD [https://datadryad.org/stash/dataset/doi:10.5061/dryad.44j0zpcb5].
