# Supplementary material for: Genome-wide identification and expression analysis of Dmrt genes in bivalves
Source: BMC Genomics. 2023 Aug 15;24:457. doi: 10.1186/s12864-023-09536-6 (PMC10428544; doi:10.1186/s12864-023-09536-6)
Supplement: Supplementary file 1 — Supplementary Material 1 [file 12864_2023_9536_MOESM1_ESM.doc]

Table S1 The genome database information for the studied bivalves

| Species | Website |
| --- | --- |
| *Chlamys farreri* | http://mgb.ouc.edu.cn/cfbase/html/ |
| *Mizuhopecten yessoensis* | https://www.ncbi.nlm.nih.gov/genome/?term=Mizuhopecten+yessoensis |
| *Argopecten irradians irradians* | https://www.ncbi.nlm.nih.gov/genome/10712?genome_assembly_id=476852 |
| *Argopecten purpuratus* | http://www.gigadb.org/dataset/100419 |
| *Pecten maximus* | https://www.ncbi.nlm.nih.gov/genome/?term=Pecten+maximus |
| *Mytilus coruscus* | https://www.ncbi.nlm.nih.gov/genome/?term=Mytilus+coruscus |
| *Dreissena polymorpha* | https://www.ncbi.nlm.nih.gov/genome/?term=Dreissena+polymorpha |
| *Mercenaria mercenaria* | https://www.ncbi.nlm.nih.gov/genome/?term=Mercenaria_mercenaria |
| *Mytilus galloprovincialis* | https://www.ncbi.nlm.nih.gov/genome/?term=Mytilus_galloprovincialis |
| *Ostrea edulis* | https://www.ncbi.nlm.nih.gov/genome/?term=Ostrea+edulis |
| *Mytilus edulis* | https://www.ncbi.nlm.nih.gov/genome/?term=Mytilus+edulis |
| *Crassostrea gigas* | https://www.ncbi.nlm.nih.gov/genome/?term=Crassostrea+gigas |
| *Crassostrea virginica* | https://www.ncbi.nlm.nih.gov/genome/?term=Crassostrea+virginica |
| *Saccostrea glomerata* | http://soft.bioinfo-minzhao.org/srog/# |
| *Cyclina sinensis* | https://datadryad.org/stash/dataset/doi:10.5061/dryad.44j0zpcb5 |

Table S2 Accession numbers of the known Dmrt sequences

| Species | Gene ID | Proein Accession Number |
| --- | --- | --- |
| Homo sapiens | HsDmrt1 | NP_068770.2 |
|  | HsDmrt2 | NP_870987.2 |
|  | HsDmrt3 | NP_067063.1 |
|  | HsDmrtA1 | NP_071443.2 |
|  | HsDmrtA2 | NP_115486.1 |
|  | HsDmrtB1 | NP_149056.1 |
|  | HsDmrtC2 | NP_001035373.1 |
|  | HsDmrtC1 | NP_001074320.1 |
| Mus musculus | MmDmrt1 | NP_056641.2 |
|  | MmDmrt2 | NP_665830.1 |
|  | MmDmrt3 | NP_796334.2 |
|  | MmDmrtA1 | NP_783578.1 |
|  | MmDmrtA2 | NP_758500.2 |
|  | MmDmrtB1 | NP_063925.1 |
|  | MmDmrtC2 | NP_082008.1 |
|  | MmDmrtC1 | NP_081867.1 |
| Macaca fascicularis | MfDmrt1 | XP_005581901.1 |
|  | MfDmrt2 | XP_045229108.1 |
|  | MfDmrt3 | XP_005581900.2 |
|  | MfDmrtA1 | XP_005581667.2 |
|  | MfDmrtA2 | XP_005543469.2 |
|  | MfDmrtB1 | XP_015291127.2 |
|  | MfDmrtC2 | XP_005589481.1 |
|  | MfDmrtC1 | XP_015299833.1 |
| Bos taurus | BtDmrt1 | NP_001071528.1 |
|  | BtDmrt2 | NP_001179302.1 |
|  | BtDmrt3 | XP_002689673.4 |
|  | BtDmrtA1 | NP_001179569.1 |
|  | BtDmrtA2 | NP_001096751.1 |
|  | BtDmrtB1 | XP_024845606.1 |
|  | BtDmrtC2 | NP_001033271.1 |
| Balaenoptera musculus | BmuDmrt1 | XP_036711655.1 |
|  | BmuDmrt2 | XP_036712720.1 |
|  | BmuDmrt3 | XP_036712559.1 |
|  | BmuDmrtA1 | XP_036712737.1 |
|  | BmuDmrtA2 | XP_036726057.1 |
|  | BmuDmrtB1 | XP_036712433.1 |
|  | BmuDmrtC2 | XP_036690564.1 |
| Gallus gallus | GgDmrt1 | NP_001095301.2 |
|  | GgDmrt2 | XP_003643035.3 |
|  | GgDmrt3 | XP_429193.2 |
|  | GgDmrtA2 | XP_015146712.1 |
|  | GgDmrtB1 | NP_001232910.1 |
| Oryzias latipes | OlDmrt1 | NP_001098150.2 |
|  | OlDmrt1Y | NP_001295953.1 |
|  | OlDmrt2A | XP_023813898.1 |
|  | OlDmrt2B | XP_004068077.2 |
|  | OlDmrt3 | XP_023813900.1 |
|  | OlDmrtA1 | XP_004079711.1 |
|  | OlDmrtA2 | XP_023810346.1 |
| Nile tilapia | NtDmrt1 | XP_013126366.1 |
|  | NtDmrt2A | NP_001266696.1 |
|  | NtDmrt2B | XP_005457139.1 |
|  | NtDmrt3 | XP_003444527.2 |
|  | NtDmrtA2 | XP_005479065.1 |
| Drosophila melanogaster | DmDSX | NP_731197.1 |
|  | DmDmrt11E | NP_511146.2 |
|  | DmDmrt93B | NP_524428.1 |
|  | DmDmrt99B | NP_524549.1 |
| Bombyx mori | BmDSX | NP_001104815.1 |
|  | BmDmrt2 | XP_004930266.1 |
|  | BmDmrtA2 | XP_021202446.1 |
| Aedes aegypti | AaDSX | NP_001345310.1 |
|  | AaDmrt2 | XP_021707667.1 |
|  | AaDmrt3 | XP_021702532.1 |
|  | AaDmrtA2 | XP_001649612.2 |
| Sagmariasus verreauxi | SvDSX | ARK36621.1 |
| Cherax quadricarinatus | CqDSX | QDM55346.1 |

Table S3 Accession numbers of the publicly available RNA-seq data

| Species | NCBI BioProject |
| --- | --- |
| *Argopecten purpuratus* | PRJNA494400 |
|  | PRJNA796071 |
|  | PRJNA418203 |
| *Mizuhopecten yessoensis* | PRJNA562987 |
|  | PRJNA259405 |
| *Mytilus coruscus* | PRJNA689255 |
| *Mercenaria mercenaria* | PRJNA596049 |
| *Mytilus galloprovincialis* | PRJNA451093 |
|  | PRJNA407964 |

Table S4 The amino acid sequences of all identified Dmrt genes

| Gene ID | Squence |
| --- | --- |
| Pm|XP_033737544.1 | MSSDDEKSDGANGMFMRAADRYPRTPKCARCRNHGVVSALKGHKRYCRWRDCVCAKCTLIAERQRVMAAQVALRRQQAQEENEARELGMLYGQTGLLQINPETVSMFPEAKQYLESNKVRDSSDEYPATKKQKIEDHREETQQETGRPDSRSSTPDGVDRPRSPDDSAGSPEPSPRSCDPPSPRSPTSKSDSKSPFDESTLAHYGGNKRQPIEMLSRIFPHMKRSVLQLILQGCGGDVVQCIEQILNSHGEASGMSSHFAGSPLITPHGISNPLGAPSLKSAFSPIASSIASAHSLNSIRNAWGSMSRGLLAMPYPPAFPGLTLGSNYSAYNGLTTDSASKPFPYTMYPYCPTKPFTASTSDKSGCLGE |
| Pm|XP_033738864.1 | MDQEADKTDGHTSRDKSVVRMTGRKLLRTPKCARCRNHGVVSCLKGHKRFCRWRDCQCPNCLLVVERQRVMAAQVALRRHQASEMTITLKDKVKTATQILQHRKLLQRNLRSLQQQSVSRDVLTKYKTKPAVYNSDGKHLPPIFNERMRKRRCFADKELELAMFERERQNEILQTKVNVGNPLGNGSSLTGLVGTDISDMTTVFPSVPRDFLQQIFPFHSSSVLELVWQGCQGNLQKAIQQIACNVPSIPSGLSSLPVPEVIHRNVFSYVKDSHYMGLEHIKEPEVDVYDGGQSIRKQHREAGCNVPKTDVSLSESKVTNVKKTLGGDSRETVVPKTARLKFSVAAIIGEL |
| Pm|XP_033737545.1 | MNPVTLYPLTEKGSRKPKCARCRNHGMVSWLKGHKRHCKFKDCACQKCNLIAERQRIMAAQVALKRQQAAEDAIALGLRACAQDHINPVMTSGPLWGPGTVSPPQGDKDGRCDDEDSIENAPKITEDSSEDDRVEASSPESINDVTETSERQKPYSPYTAVTRARDLHSTTPFLPGHLNNLEILERVFPFQRKSVLELVLQGCNGDLVKAIEQFLSTQDNLMTQHTTPRQKQDFRSHPYFGQLPLHTIKSLNSKMPNGSSMRSAFTPFSSPAPMAHTGLHSAFTSPVSTMSCDALRAQMFPSGIRSGDVLPPTSQFPYPSFSHLTSGPLPGFMSSPFSLYPYRTGITDMNCFRKTAEKASERAALQESNQNAENWDESAKERDVE |
| Pm|XP_033733655.1 | MSSKKESKHALGLSPLRSPKCCRCRNHGIITVLKGHKRFCQWKNCTCENCLLLMKRQQNSKEQIALRRLWKQEEDMGLVAPIPVDTDSLQVLIHRYPHYDVEKLSAILKSSDGDTQKAIEKIDAAMNRALDNITTEAKPGLTPTCAGDMRVTPGHVGMGFIGFQGYNSGFHTPLPSPHPSVNPLSPRASMPMYPRAPYMYPYNPPSRLFPATQPDTTYTGDFHGPMSRSGQSAQDFPTSRNVFYSECQSSMESLIGYPKASEVGVFQPRPSTANSDTEGNLVIDMKDEEGACAEGLLANYSSQ |
| My|XP_021377274.1 | MSSDDEKSDGASGMFMRAADRYPRTPKCARCRNHGVVSALKGHKRYCRWRDCVCAKCTLIAERQRVMAAQVALRRQQAQEENEARELGMLYGQSGLLQINPETVSMFPEAKQYLDTSKVREAPDDYPASKKQKLEDPREEFQLDAARPDSRSSTPEGVDRPRSPADSAGSPEPSPRSCDPPSPRSPASKSDSKSPFDESTLAHYGGNKRQPIEMLSRIFPHMKRSVLQLILQGCGGDVVQCIEQILNSHGDANVMSSAFSGSPLITPHGLSNPLGGPSLKSAFSPIASSIANAHSLNSIRNAWGNMSRGLLAMPYPPTFPGLTLGGNYSAYSGLTTDSASKPFPYAMYPCCPTKPFTASASDKSGCLGE |
| My|XP_021368788.1 | MIEPEEEKQEGHTDMLRDKSVGRVSGRKLLRTPKCARCRNHGVVSCLKGHKRFCRWRDCQCPNCLLVVERQRVMAAQVALRRHQASEMTITLKDKVKTATQILQHRKLIQRNLRSLQQHTLSRDVLSKYKNKQTIYNADEKYLPPIFNERMRKRRCFADKELELAMYEHERQHEILQTKINVSNSSGTGSSLTGFVGTDTIDMTSTVPSSSRDVLQQLFPFHSSSVLELVWQGCRGNMKKAIQQIVCNVPCIKSCHPSVPVPEIIHDSVFSFVKDSHFTRLEQEKDPKVAYGDRNISWRQQSETDCSTKRKELSPLGTKNTTGHGKSAVCATREAAASKPGRLKFSVAAIIGEL |
| My|XP_021377273.1 | MNPVTLYPLTEKGSRKPKCARCRNHGMVSWLKGHKRHCKFKDCACQKCNLIAERQRIMAAQVALKRQQAAEDAIALGLRACAQDHIHPVMTSGPLWGPGTVSPPQGDKDDREDDDDSIDNAPKITDDSSEDDRVEASSPESVNDVTDLSERPKPYSPYTSLTRTRDLPSTTPYLPGHLNNLEILERVFPFQRKSVLELVLQGCNGDLVKAIEQFLSTQDNLMAQQTTPRLKQDFRSHPYFGQLPIHTIKSLNGKLPNGSSMRSAFSPFSSPAPMAHSGLHSAFTSHVNTLSCDALRAQMFPSGMRSSDVLPPPSQFPYPSFSQLTSGPMPGFMSSPFSLYPYRTGLTDMNCFRKATEKTSERAALQENNQNVENWDESAKERDVE |
| My|XP_021353714.1 | MSSPKETKRATCLSPLRSPKCCRCRNHGIITVLKGHKRFCQWKNCTCENCLLLMKRQQNSKEQIALRRLWRQEEDMGLVAPIPNNTDSLQMLIHRYPHYDVEKMGTVLKSCEGDTQKTIGKIDAAMNKALDTITHTKLDLTPTCTGDLRVSTGNVGMGFIGFQGYNHGFSAPLPPPHTNVSPLSPRASMPMYTYPRTPFMYPYNPAPRFFQTGQQDITYNGDFHNTLPVPRSGQSAEDFPTSRNAFYSEGQSSMESLIGYPKVSEVNVFQPRPSTTESEAEGTLVIDMKDEESACAEGLLTNYSSQ |
| Aii|evm.model.Contig349.42 | MSSDDEKSDGTNGMFMRAADRYPRTPKCARCRNHGVVSALKGHKRYCRWRDCVCAKCTLIAERQRVMAAQVALRRQQAQEENEARELGMLYGNTGLLQINPETVNMFPEAKQYLESSKRETSDEYPASKKLKLEDHRDDLQPEATRPESRSPTPDVMDRPRSPADSAGSPEPSPRSCDPPSPRSPASKSDSKSPFDESTLAHYGGNKRQPIEMLARIFPHMKRSVLQLILQGCGGDVVQCIEQILNSHGEAGGMSSAFAGSPLITPHGISNPLGAPSLKSAFSPIASSIANAHSLNSIRNAWGTMSRGLLAMPYPPAFPGLTLGSNYSAYSGLTSDSSSKPFPYSMYPYCPTKPFTASASDKTGCLGE |
| Aii|evm.model.Contig349.40 | MNPVTLYPLTEKGSRKPKCARCRNHGMVSWLKGHKRHCKFKDCACQKCNLIAERQRIMAAQVALKRQQAAEDAIALGLRACAQDHINPVMTSGPLWGPGTVSPPQGDKEGRCDEEETNENTPKMTEDSSEDDRVEASSPESVNEVTETPERPKPYSPYTSLTRPRDLPSATSFLPGHLNNLEILERVFPFQRKSVLELVLQGCNGDLVKAIEQFLSTQDTLMTQQTTPRLKQDFRSHPYFGQLPLHTLKSLNSKLPNGSSMRSAFTPFSSPAPMAHTGLHSAFTSPVNTMSCDALRAQMFPSGMRSSDVLPPSSQFSYPSFSHLTSGPLPGFMSSPFSLYPYRSGITDMNCFRKTSEKASERAALKESSQNVENWDESAKERDVE |
| Aii|evm.model.Contig6.279 | MSTKKETKPAVGLSPLRSPKCCRCRNHGIITVLKGHKRFCQWKNCTCDNCLLLMKRQQNSKEQIALRRLWKQEEDMGLVAPTPVSTDSLQILIHRYPHYNVDKLGAILKSCDGDTQKAIERIDAVTNRALDAITTEPKAGMTPSCAGDMCVSPGNTAGMGFVGFQGYNSGFHTPLPPPSSAVNPLSPRASMQMYPRTPYNMYPFSPPRFYPPTQQDISYNGDFHGPTPRPGHSAQNFQTSRNAFYSECQSSVESLIGYPKVSEAGVYQSRPSTASSETEGNLVIDMKDEEESVCAEGLLTSYPTQ |
| Cf|CF57815.1 | MNPVTLYPLTEKGSRKPKCARCRNHGMVSWLKGHKRHCKFKDCACQKCNLIAERQRIMAAQVALKRQQAAEDAIALGLRACAQDHINPIMTSGPLWGPGTVSPPQGDKDGRGEDDESIDNAPKITDDSSEDDRVEASSPESVNDVTEVSERSKPYSPYTSLTRARDLPSTTPYLPGHLNNIEILERVFPFQRKSVLELVLQGCNGDLVKAIEQFLSTQDNLMTQQTTPRLKQDFRSHPYFGQLPIHTIKSLNAKLPNGSSMRSAFTPFSSPAPMAHTGLHSAFTSHVNTLSCDALRAQMFPTGLRSSDVLPPPSQFPYSSFSQLTSGPVPGFMSSPFSLYPYRTGLTDMNCFRKATEKTSERAALQESNQNAENWDESAKERDVE |
| Cf|CF58131.2 | MSSDDEKSDGANGMFMRAADRYPRTPKCARCRNHGVVSALKGHKRYCRWRDCVCAKCTLIAERQRVMAAQVALRRQQAQEENEARELGMLYGQSGLLQINPETVNMFPEAKQYLDTSKVREAPDDYPASKKQKLDDPREEFQLDAARPDSRPSTPEGVERPRSPADSAGSPEPSPRSCDPPSPRSPASKSDSKSPFDESALAHYGGNKRQPIEMLSRIFPHMKRSVLQLILQGCGGDVVQCIEQILNSHGDVNGMSSSFAGSPLITPHGISNPLGAPSLKSAFSPIASSIANAHSLNSIRNAWGSMSRGLLAMPYPPAFPGITLGSNYSAYSGLTTDSASKPFPYTMYPCCPTKPFTASASDKSGCLGE |
| Cf|CF3811.22 | MIEPEDGKLEGRTDMLRDKSVGRMSGRKLLRTPKCARCRNHGVVSCLKGHKRFCRWRDCQCPNCLLVVERQRVMAAQVALRRHQASEMTVTLKDKVKTATQILQHRKLIQRNLRSLQQHTLSRDVLSKYKNKQTVYNADEKFLPPIFNERMRKRRCFADKELELAMFEHERQNEILQTKINVSNSSGTGRSLTRFVGTEAIDMTKTVPSSSRDVLQQLFPFHSSSVLELVWQGCHGNLKKAIQQVVCNVPCIKSCLPSVPVTEIFHESMFSFVKESDFTRQEQEKDPDVLYRDRDVNLKQRSETDLSTKSKVLSPLETKNTTLHGNSTVCANREAAASKPGRLKFSVAAIIGEL |
| Cf|CF42417.14 | EQRSSLTLSHGIKKSRETWVPYPVQKVVKQPHKNLLKIIFPVIDKLMSSPKETKRAACLSPLRSPKCCRCRNHGIITVLKGHKRFCQWKNCTCENCLLLMKRQQNSKEQIALRRLWRQEEDMGLVAPIPNNADSLQLLIHRYPHYDVEKVSAVLKSCDGDTQKAIGKIDEAMNKALDNITHTKLKSDLPPECTGDLRVSSGNVGDLRVSSGNVGMGLIGFQGYNTGFHAPLPPPHTNASPLSPRASMPMYPRAPFMYPYNPAPRFFPAAQQEVHNVSYNGDFHGTLPVPRPGQSAGDFPASRNVFYSEGQSSMESLIGYPKVSEVNVFQPRPAATESEAEGNLVIDMKDEESACAEGLLTNYSS |
| Cg|NP_001295834.1 | MSSDEEKGDSHGSVFMRASDRYPRTPKCARCRNHGVVSALKGHKRYCRWRDCVCAKCTLIAERQRVMAAQVALRRQQAQEENEARELGMLYGPNGLLQLNPETITMFPDAKKVVDTSGSDREDGPATKRQKLDSSRTDSPVSRCSSEDMNERTHSPADSTSPPTSPKLADPPSPSDDKPEPFPKSPFEEGLLAGNSKKNPIEMLQRIFPHMKRSVLQLILQGCNGDVVHTIEQVLSNHGTDQSSATSTSSSSFMPHPGLVSTMTNSSLRSAFSPISTLANAHTLNSMRYAWGSMGGRGLLAMPYPPVLPGLTLGAAYSNYSGLNSSSNGAKPFHYAMCPCCTTKPFPSSNSEKSSYIAE |
| Cg|XP_011427033.2 | MNPVTALYPVTEKGTRKPKCARCRNHGMVSWLKGHKRHCKFKDCNCAKCSLIAERQRVMAAQVALKRQQAAEDAIALGLRCATEGSLPIMTQGPLWGPGTVSPPEDKDKKGTDSDSDDLDDNSSDDNEPATTVVNTAPEPTNSRPDTSTPDSGEEMDPARVKPSVPSTYPHSLLPPNAFLPGRLSNLEILERIFPFHRKSVLELVLQGCNGDLVKSIEQFLSSQESMLSSPHIKSDLRPHPYLQPFQYGSAMKSLNGISRLSQNGPGSAFTPFSSAGPFNPSAIHSAFQSHLSNFSTDSLRPTYMAGSTRPDIFTPNNQLTYPRLHPLTSAPFPGFFSGSFPLTPYRLDMSDVSSCHKPHSDKLTGKGLSPEKGHEADMWDDASRERERE |
| Cg|XP_011441049.2 | MSGNIIIDSELSTRRCNKCRNHGRLVTLKGHKPFCQFKNCSCKACTQLEMKKLSTALRRKEKIARDSKIDPLFDTLCQPPVGPCLVRYQPYKAMRIPCNNPSSGSMVESCMVRYQPHRAPIKSQCYSPPDTLVEPSITLSLPSLHAASNSKTSSTSYKTEMRRYSNITKSIMPTSKTTMDTSAAEPSIEVLKQNLLVSTNNVIQQVPRTYWITQSTNTRHAIQPPRGSQAAGISHPCFSLSSSFHHAPACSVQSMPWLCEPTYTTLKPVSHVGLSYSADGAFWMQQEQGSQSREAGAMYRDTN |
| Cv|XP_022319926.1 | MSSDEEKGDSHGSVFMRASDRYPRTPKCARCRNHGVVSALKGHKRYCRWRDCVCAKCTLIAERQRVMAAQVALRRQQAQEENEARELGMLYGPNGLLQMNPETITMFPDSKKVHETSGSDREDGPAAKRPKIDSSRTDSPVSRCSSEDLNERTNSLADSASPPNSPKLPDPPSPSEEKSEPFLKSPFEESLLAGGSKKSPIEMLQKLFPHMKRSVLQLILQGCNGDLVQTIEQVLSNHGHGAEHSSSSSTSSSSFLPHPGLVSTMTNSSLRSAFSPISTLANAHTLNSMRYAWGGMGGRGLLAMPYPPVLPGLTLGAAYSNYSGLNSSSSGAKPFHYAMCPCCTTKPFPSSPSEKSSCIAE |
| Cv|XP_022317913.1 | MNPVTALYPVTEKGTRKPKCARCRNHGMVSWLKGHKRHCKFKDCNCAKCSLIAERQRVMAAQVALKRQQAAEDAIALGLRCATEGSLPIMTQGPLWGPGTVSPPXDKDIKGQDSECDDLDGDSSDDNEPVPKVVNTVPESTKPRPEXSTPDSTDDSEPARPSKVPAPATYPHSLLPPNAFLPGRLTNLEILERIFPFHRKSVLELVLQGCNGDLVKTIEQFLSSQESLLSGQTPHIKSSDLRPHPYLQSFPYGTTMKSLNGISRLSQSGPXSAFTPLSSAGHFNPSGIHSAFQSHLPNFSADSLRPQFMSGAARPDIFAPNNQLTYPRLHHVTSAPLPGFFGGSFPLTPYRLDISDIXSCHKPLSDKLSGKGVSAENGRDSDVWDETSRERERE |
| Cv|XP_022333988.1 | MMMSDQSSSVNDVAMRRCSKCRNHGVLVRLKGHKPFCRFRDCSCMACCRLEEKKVVTRIRKMQLATANVQTGPGQVDPVPEPPQQRPCSVRYHPYTHKIAKSQVDKPPQSLKETQVPPGLTPNHKPTSYYINNLQTKTLWEKTCDSSKMMQYTNITKTIIPKEPTPVTGPNVAKARKMEAQQPTYCMPGPNPATGASVAKARKMEAQQPTYCMPGPNPTTGASVAKARKMEAQQPTYCMPGPNPATGDNIGRARNLEPQQPTYCMPAQLSSSNLTTQPSTTQQATWTGTKASEIYRTQCMDCPGPFQTTINTHFPTYTLLNPVSHVGLIFSSENTFWARQEQDMLIFNQQWNVQHTNY |
| Dp|KAH3782801.1 | MTNGTDDDISVVDAKSTGAVMIPGPMFLRTIDRFPRTPKCARCRNHGVVSALKGHKRYCRWRDCTCAKCTLISERQRVMAAQVALRRQQAQEETEARENVMYASQTSGLTAGREMTPAFHEGIRSRGYACSTSSYDGSDNDEPPAKRLRVSDIYTSGLREAPRSPADSRGTSSPHSTASSGSPIPEHFNHSADDDIVRFDDETGNRKSVDMLCRIFPTHKRHVLQMILHGCHGDVVQAIDQLFKTNNDERIPDSTMLHYATMPISGYTPRIPNIETSFKSAFTPRSISGAGFAVAPTLNPFSPYTWHSGSLPPRGLLSYPYDGALLAGLHSNLGAYASLGSTSAPNKPVMYSSYPFSHFTQDRPAELDSR |
| Dp|KAH3696108.1 | MRILFSYNLVEVKSDFCGEKVSRLNSTENTELRLKRHILESVVLNIKDMSSSSDEERHGADGGNMNGSADGGLLRSSSDRYPRTPKCARCRNHGVVSALKGHKRYCRWRDCMCAKCTLIAERQRVMAAQVALRRQQAQEENEAREMGVLYGPSGLLQINPDCASMFPDAAKSVDGTQKVNADDEPETDSPPSSKRQKLEQNEESGRLSVSSDDSRSISPSYSDDRRYHGDRPPSTSKTPHSGPLEELPSVYDRNGRLSENWLLNFAQSQNGSKKTQQPIDVLCRIFPQKKRNVLELILQGCGGDTVQAIEQVVATQRQEESAASGMLYAGLSHNHMTNHLQSSIFKSAFSPLPTFSAANALSTMRYAWGGAAGNRLALTMPYQHFLPGLSMGSSFGYGPLSNSADKISPYSMYPFWAGKPFGTKEVEKSPGCVSD |
| Dp|KAH3699546.1 | MTSPALAVNGYDVSASSRSGTLMDGRTVDRMPRTPKCARCRNHGVVSALKGHKRYCRWRDCLCAKCTLIAERQRVMAAQVALRRQQAQEESEAKDLGILYGPGGLLQINEGTADEDEVRRKRTLKTGGHVDDDISPRHIKTEPIIRIPADNVNRNRKIVAEDRPSKSPGKPSEISQQQRDYGLSKIKVDDEKSSNVDSWFQTWSGSAVKNLYSVYGGDMSKYVGFFNKSLASQRKIDSLYPPPLMFTSGFHSDIDASTGSGMLFGQFGSNYSQMTSSLEAMRSPWNIYARFPGISFQHYLGAESSRASDANKHPLAGNPQTSQCSGDTE |
| Dp|KAH3721156.1 | MNAMSHLYPVTEKGTRKPKCARCRNHGMVSWLKGHKRHCEFKDCTCAKCNLIAERQRVMAAQVALKRQQATEDAIALGIRACAHDGGVPIMTQGPLWGPGTVHPPETNKEREERMNRENIMRNHTGINFVYNKSIIIIIVYYYYYYYYYYYYYYYYYYYYYYLC |
| Mc|CAC5398878.1 | MSSDEEKGDSANSLFMRASDRYPRTPKCARCRNHGVVSALKGHKRYCRWRDCVCAKCTLIAERQRVMAAQVALRRQQAQEENEARELGMLYGPTGLLQLNPETVTMFPDTKKFQTPISTEETPAKRQRLDKDTDHSPAGNSPSSSPDINESSRSSPGNYRHSPIPSPKSYETPSPTADSAFRKSNEEINNLNSLNMLCRIFPHMKRSVLQLILQGCGGNMVQSIEQVLSNHCSEQTDSGIHAPNPLMSPLSSPLTGTNFKSAFSPISTLANAASLNAMRYAWGGIGGRGLALAMPYPPMLPGLSVGANYSSFEGLSSNGTKPFHYSMYPCCPTKPFPQSSTDKSNCLTD |
| Mc|CAC5404148.1 | MDITKCVISVITPKMEDNQITSKLGAKSLRTPKCARCRNHGVVSCLKGHKRFCRWKDCQCPNCLLVVERQKVMAAQVALRRHQSSESPNKVTQKVVNATRVLQERRMMHRSLRHLQQRVVSRDIISNIHQRIFQNNPTKNNDKMQLNERIRRRRCFADKDLDTASVGVITKFSEPIDTKRDYYSLNSIKVIPSQISMLRHLFPWSNSNILELVLQSCHGDLDTAIYHLKHRTLRQQEHADFQSSIKNINAYVLPFDNNLSTRKDGVPEKSDGAIYGLKCGAFTRFTNLSVEKEECFDNSNSNHKLNLDFLKTEKLKSTSADLKFSIASIIGEN |
| Mc|CAC5360634.1 | MNPITALYPVTEKGTRKPKCARCRNHGMVSWLKGHKRHCKYKECACAKCNLIAERQRVMAAQVALKRQQAAEDAIALGLRACTSEHGIPVMTQGPLWGSSTITPPEKSKTDEETKEKGTESGNERRIDSSTPESVDSGDKYHQEETTQYAPTTFLPGRLNNLEILERVFPFQRKSVLELVLQGCSGDLVKAIEQFLSTQENMTAQQTCSTIKKDFRHHPYLGSHNFQSTLRNYDMNKLSPLGSMRSAFSPFSSPSALSFNGLHSAFSQNMSPSNSYGTSFYSPHLRSMDLFSSPAQYPYSRYASLNSSQLPGFFASPFCVQPYKTGYGGISLNTENRDKNNKSPTSETGNGSDSWDDHGKDKD |
| Mc|CAC5397186.1 | MPPDSKQRQRECQKCLAHGVHVRFKGHKFGCFFRDCQCQRCQVVSIKRQKNKLVTAQKRRLEMDKVKKFETEQANGKGSGEISDSEENQLIIDESSNQQQIQLSDRRAVYCRRCQIHGVKNLVRGHKDSCPYRGCWCTECRIINSQSSVCSHCGFYPPVTPYPRPYPPSLNWLLHQQNNTACGEGNTSSSNYSSNLGYKSQVNYVQPTTLSLPYNSTPGYMQGPQHDIPHSTVPYETPASQQDGNLSL |
| Me|CAG2209978.1 | MSSDEEKGDSGNSLFMRASDRYPRTPKCARCRNHGVVSALKGHKRYCRWRDCVCAKCTLIAERQRVMAAQVALRRQQAQEENEARELGMLYGPTGLLQLNPETVTMFPDAKKFQSPISTEETPAKRQRLDKDTEHSPAGNSPSSSPDINESSRSSPGNYRHSPAPSPKSYETPSPTAESAFRKSNEEINNLNSLNMLCRIFPHMKRSVLQLILQGCGGNMVQSIEQVLSNHCSEQTDSAIHAPNPLMSPLSSSLAGTNFKSAFSPISTLANAASLNAMRYAWGGIGGRGLALAMPYPPMLPGLSVGANYSSFEGLSSNGTKPFHYSMYPCCPTKPFPQSSTDKSNCLTD |
| Me|CAG2252366.1 | MEGNKISKLGSKSLRTPKCARCRNHGVVSCLKGHKRFCRWKDCQCPNCLLVVERQKVMAAQVALRRHQSTESPNRVTQKVVNATRVLQERRMMHRSLRHLQQRVVSRDIISNINQRIFQNNPMKNNNKILLDERIRRRRCFADKDLDTASVGVITKFSEPMDTKRNDSSLNFIKVLPSKISILRRMFPWSNSNILELVLQSCHGDLDTAIYQLKLTTLRQQDYTDSQRSSKNIDAYILPFDNNLFTGKDGVQEQSDGSIYDLRCGVFTRFTNMSVKKEECFDNSNLNHRLDLDYLKTEKLKSTSSDLKFSIASIIGEN |
| Me|CAG2226664.1 | MNPITALYPVTEKGTRKPKCARCRNHGMVSWLKGHKRHCKYKECACAKCNLIAERQRVMAAQVALKRQQAAEDAIALGLRACTSEHGIPVMTQGPLWGSSAITPPEKSKTDEEKQEKGSESGNERRIDSSTPESVDSGDKYHQEETTQYAPATFLPGRLNNLEILERVFPFQRKSVLELVLQGCNGDLVKAIEQFLSTQENMTAQQTCSTIKKDFRHHPYLGSHNFQSTLRNYDMNKLSPLGSMRSAFSPFSSPSALSFNGLHSAFSQNMSPSNSYGTPFYSPHLRSMDLFSSPAQYPYSRYASLNSSQLPGFFASPFCVQPYGKTGYGGISLNIENRDKNNKSPTSETGNGSDSWDDHGKDKD |
| Me|CAG2232556.1 | MPPDTKQRQRECQKCLAHGVHVRFKGHKLGCFFRDCQCQRCQVVSIKRQKNKIVTAQKRRLEMENVKKIETEQANRKESGEISDCQSSVCSHCGFYPPTVTPYPRPYPPSLNWLLNQQNSTSCGGGNTSSNNYSSNLGYKPQVNYVQPATPSMPYNSTPGYMQGPHHDIPHSTVLYGTPASQQDGNLIL |
| Mg|VDI24477.1 | MSSDEEKGDSGNSLFMRASDRYPRTPKCARCRNHGVVSALKGHKRYCRWRDCVCAKCTLIAERQRVMAAQVALRRQQAQEENEARELGMLYGPTGLLQLNPETVTMFPDAKKFQSPISTEETPAKRQRLDKDTEHSPAGNSPSSSPDINESSRSSPGNYRHSPAPSPKSYETPSPTVESAFRKSNEEINNLNSLNMLCRIFPHMKRSVLQLILQGCGGNMVQSIEQVLSNHCSEQTDSAIHAPNPLMSPLSSSLAGTNFKSAFSPISTLANAASLNAMRYAWGGIGGRGLALAMPYPPMLPGLSVGANYSSFEGLSSNGTKPFHYSMYPCCPTKPFPQSSTDKSNCLTD |
| Mg|VDI42071.1 | MEGNKIISKLGSKSLRTPKCARCRNHGVVSCLKGHKRFCRWKDCQCPNCLLVVERQKVMAAQVALRRHQSTESPNRVTQKVVNATRVLQERRMMHRSLRHLQQRVVSRDIISNIMTKLKASINQRIFQNNPTKNNDKIQLDERIRRRRCFADKDLDTASVGVITKFSEPMNTKRNDSSLNFIKVLPTKISILRRMFPWSNSNILELVLQSCHGDLDTAIYQLTLTTLRQQDYTDSQRSSKNIDAYILPFDNNLFTGKDGVQEQPDGAIYDLRCGVFTRFTNISVKKEECFDNSNLNHRLDLNYLKTEKLKSTSSDLKFSIASIIGEN |
| Mg|VDI32052.1 | MNPITALYPVTEKGTRKPKCARCRNHGMVSWLKGHKRHCKYKECACAKCNLIAERQRVMAAQVALKRQQAAEDAIALGLRACTSEHGIPVMTQGPLWGSSAITPPEKSKTDEEKQEKGSESGNERRIDSSTPESVDSGDKYHQEETTQYAPATFLPGRLNNLEILERVFPFQRKSVLELVLQGCNGDLVKAIEQFLSTQENMTAQQTCSTIKKDFRHHPYLGSHNFQKNRKFRKDTSRLSGEYQGAKPIPFKWTTPYGTPPNTYLTFHTSLSNFATSINWLHYNAKKKPSSHPTRDITVKNGAIFFPRVLRPWTSASCTGIIIVNHFQFSFILTFFYLNCPGIVNCNYCSYVLKGPEFAPIQSHSNRIWNFTGNHCEKIMDDPTIAFNHAAFYDKSKLHKLENNPNYVGNDIPVYWTNFVGFSTIETSMAAKYLQPPDTRDPDHYVTAFRVGLIVQKLTVSFYKNGIFESSQVKECSGMDRDLPVDSKHCSNQFILSYFPTTLHNDKIMFAVSAENGGYLDVHNRETDGVNRYYYEGNTLVREFEFHWDLVNPYHCSVTQGLGCDAFASVTNDITDNPNIDLSFGGWADDLSGLSYYDYDVYDLGHNGVDLIDGMSVVISQTKIHVDNRANTFIVPRPGAYSLHFFAFDKAGNYKTGRTLFLFDDQSVVDKYEDKQTICSTSSQNTSYQWVVTDTDTLQIIWTDRFINSRHKDNRWLNEVQTFSPSAETSIYEDLYGSRTNKLINNQHGIVDFQVSFQVHSPLLKDSRPLTSVSDIHNQYDILKVNWADGDRLTSTVRALDVLGKFNEDTIIIYRDVTPPVIENLWLTRGDRVNISVHSIEDFTQMTIEWEAYDDHSGLDSIYWKLYDKYGGTEVLHGHEDIITQGGADALEECENMYKSYARGANCYCTPFMGCYHRHFQVKPEIKAHGGLVHNLYKGVHDYDYHIDISVTNKANITTLLTKKITIDISPPHAGVVHDGIVGSPEIDYQQELTIRAYWDQFFDRESGVFFYQYIVGSNCANKEDFDLELTHPNVVETYDTFASETLTSEGTYYFTVIAYNRALEPSDPVCSDGVTIDTSVPSIEQVTVENAIIMGGLIKDTSKYYILGSDRNRRLISNPTTECIDKATLIPDIDLYPLEQYDNGTVVEVNGTVFCQNSTGAPSNIGITLSKSSMMEISWIPVLVPGQIYDYEVGLSSTSGSSAPDLLAFQSTKQHAHYLIMHSNIPDGARFFIVIKTISKSNKEGITSIGPFFVDTTPPDFTGSISLTLSGDYLLATWASDAFVDSEEFYELDYEFSVGHEKYSTDVQGFIPLKEGGGCLITSSPICTAVKIIDLDWYLHGHHTYYISIKVTNTAGLTAIQASSPYIHDVQLPAEGFVLDIDSQDIEDIDFQADKTSIASRWSGFVHPHLDVTYRISIGSTKGGSDIISLKDVGSVTAHKETGLSLEPLKKYFVTVTAVTSAGSIRVSSDGVTIVTENAALPGVMIYDGKPCNMTDFTLNHHEEDHRFPCPDDIDVQMSLNSIKAYWTVPEDIQTYTFDALFAIERRSSLGDMWFPFHGFDYVSTVFDITVDDLTLSPGFMYRVVLKLCANTICFQTISTDGVMVMSSPPATGAITLQHLNLTAGGGTEKLLVTFDQFYDPDIENITEKYDAIPTYEYAITDNSMLGKMYTPWTSITSETTDNHKVSFVVPLTGEMDFSRCKRFTIRGYNKAKHYSIVSTEIKDCAAYNPILINPNIVIDAVGQPDLVDGVGRTIYLTKNDYWTEADQDYTPYKNVISAVWPTLRHRSYTYAVLNAKTIDVTTYYRQINQLTLKDPCDHPDAIKCGSTNSEFMNVEFGDGELVHGTRYTVCVHADPRVIEYEMWTDVLPEVNSCSDGIIVDLTPPVEGRVWIGNIPTVTHQTVTTDMYVNWDGFYDVEEYNNGPHSTGIKEYILGIGTTSGGNDVFPFENVGTVQHKALHGFNLQNGYKYYATIKAVDFANRETTVMSDPVIVDITPPDKSNDPITITNLHITSTTEIEACWKNVFTDLESGIDYFLWSIGSEPGYVDIMSYVTVFEEECDTTDKNNPLDLLDGHYYYINVRAFNKAGLSSLATSWAFQVDTTPPTPGHVYDGDNTVLTGDVKDIDYQTETKVIHVYWEGFHDTHSIIQEYYVSVGTCPQCEDILTEQAVGITNEFTLKDIHLGTGLRYYTTVTACNTASMCTSVTSDGVIIDNSPPIAGHVQDGTGFYDTQYQSMRTYISAKWNGFDDPQSGLQKYEWRAGTSIGGDDIVSVTELHLTEVLALNDLSLSLPVNQKIYLTVRAYNRAGLWTESSSNGFIVDITAPVVSTSLTFAKDYGINGLTQIYRDSMKVEWDIDDPESFIERQYLSISSHIGGEFNLSSIHVNGIARWYVITGLDLHDGVTYYVTLISCNGAEICITSTSPGMFVDSSPPSRGMFAINTDHAVNSDLSRHSDGWMTWTSASLYLAWLGFSDTHSSINHYKVTVGSSYMATDVGKKPDAKYIHSTANEDNGDEGKVQLFKVQTKGLINYDILYVTIWAVNGVGLRSDLIYSAFRRVPGGALELIRRCDSMSCEGHCVCAPQDQKCPSSPSLNSCTDISSNNSNSILLVMDSNGYSSTDLSFTSSANILRGQWIKSQSQGLAPLWYEWSVGYTGFSTPTGIYNLVDEKNWQDGGQFTDGVYITNRDVNLVEHSSYSFYVRVWYTVDSYAVFKSDGITVFSQTPPVASVRGKVVTEKTLGTNVKDQDFIQTGRPMVIDWSGKFLEAENLVQKYKIYLSTYPGGHDIWTGSDELPGSATGYNISRLSLVPCALYFTNVVAYTYSGVHTTVTSDGFIVDVSPPVSGIVYDGIGRSDLEYQNSSDFISARWHGFIDTDSGIKEYRWCVGTMVNPVECSVIGWTNVGLHVSVARNLSSSITQGSKIYSKVYAIDNVGLRSDTAISDGVVIDTTKPVSEKFLHADANLVSNPSFENTSGSELMWEDVNSTDICMLSYIYYPSSWSPEPLACLAVVSSDVNLAKDGRSFLFIRGSAHQRLENIIAGTLYKVSFFSSHLPIWDSVAANKQGFIQLGDHSEIFLIYTKAYRRDNHGDSSREEVSWHKHTYYIKANEDNVNLTIGSADQTTGLLIDDLSVQEVTLDSNDISGGHILGHVVYIHEWGSIHGSWSFSDPESPIIDYSWAIGYAEGGTQIQPFRSVGVMNFGYNNNITLVHNVYIYITVIATNTAGLRGVAYSDPILVDLTPPDINFINDGLSIDEDSWILNEVSAKWEVEDLQSGIKECKWAIGYLPERTDLQIYTTVSGNTGFREFDYSVLEGYTVYSTLSCENNAGLSSVKSTDGVRITEKRPSTDHAEVEVVGISSTQYNPQTGFQGVTDNIRLKWSGFTDNVGIDAYVVDIGGDSLELMFFPTEQDLMYTHFSQMSIASDIQQKLSIQAVNKVFLRSDKVRKDLLVYLDTPQVDATKTVSVLWTENKFMASWEGIFLSDHQLYYEVSAGTHDSGVNILQWQYTNQTSVTFGLPGSVQASSGLKVHITIGAVSTGGFYNFIKGTVILP |
| Mg|VDI03798.1 | MPLDSKQRQRECQKCLAHGVHVRFKGHKLGCFFRDCQCQRCQVVSIKRQKNKIVTAQKRRLEMENVKKFETEQANRKGSGEISDSEENHLIIDESSNQQQNPISDRRAVYCRRCQIHGVKNLVRGHKDSCPYRGCWCTECRIINCQSSVCSHCGFYPPTVTPYPRPYPPSLNWLLNQQNSTSCGGGNTSSNNYSSNLGYKPQVNYVQPATPSMPYNSTPGYMQGPHHDIPHSTVLYGTPASQQDGNLIL |
| Mme|XP_045157593.1 | MSSSSDDERGQAENTSEPNGGDGVFMRSASDRYPRTPKCARCRNHGVVSALKGHKRYCRWRDCMCAKCTLIAERQRVMAAQVALRRQQAQEENEARDMGVLYGPSGLLQINPECATLFPDATKVLDSTKARHDVDVTAERPADDMPAAKRQKLESRKEESGRLSASSDDSRSVSPSYSDDKRCHDNTRASPIQHSPSAYDDIATFYERNGRLSENWVLNLAQNSSKKQQHPIDMLCRIFPHKKRNVLELILQGCGGDTVQAIEQVLTTQREEEKNTPGLLYPSNPAYPSLSTPLQNSVFKSAFSPISSLSAANTLNTMRYAWGGAAGRGLAMTMPYSHLIPGLSMAPTFGYGTMGPAGDKLSPYSMYPFWTTKPFSSKEVEKTSGCVSD |
| Mme|XP_045159713.1 | MTNTTDAERTVDSENVPANTGAVTIPGPLFLRSMDRYPRTPKCARCRNHGVVSALKGHKRYCRWRDCVCAKCTLISERQRVMAAQVALRRQQAQEENDVREMGYLYATQTANMSLGHETTGSIYPEARDRVGSTGSYSDTSDSEQPPAKRMRLEESYTTTLGIALRDSAHSPDSRGSVSPHSTTSSSESPVPETFHSHNNLDDSSDGTNKSVDMLCRIFPHMKRHVLQLILHGCQGDVVQAIDQIFNSQSDTESQSKPTMESVPLVASPAYTPTTLDSSIKSAFTPRSISGLTASQSLSPLRYSWGGMPPRGLFSVPYQSMLPSLASSFHPYSGLPQSPPVSKPVMYSSYPFSHFTQDRPADLLESR |
| Mme|XP_045156965.1 | MDKGNIADEKGQQVINDVKTPRNDKTGPRRLLRTPKCARCRNHGVVSCLKGHKRSCRWRDCTCPNCLLVVERQRVMAAQVALRRHQASENAELMKEKVEIASSLLSHRKSVQRSVRQLKLGTIQHGGGTTGKHLHRSSTKPDALSDRLRKRRCFADEELDRTISIATRGFERTPPGNYSVGSTSLQRNIPVTERVLNNTIPQTVNLNRILPRASSCFNVPIHVQKQTRCTDSAGEYMREMLTGTKLRKLEFVWPNGDGCAETMFQNPLLFVPGQTPWSGFSARLPNWKTAFIQSYKEKDVHPSTLVKKRENKEKAKSREFPVKLSNISCSSECQNASSKLQDLKFSISSILGLKE |
| Mme|XP_045157038.1 | MNSLSHLYPVTEKGTRKPKCARCRNHGMVSWLKGHKRHCDFKDCTCAKCNLIAERQRVMAAQVALKRQQATEDAIALGIRACAGTESALPIMTQGPLWGPGTVAPPSSNAERENNERLSRSQPSPDSRSEDEDDVSVCSDEDQHFTKPEDCHRRIPTPNAADKQDGKCDERDSIIKSNIIKPAAFTPGRLTNLEILERVFPLHRKSVLELVLQGCNGDLVKAIEQFLSAQDTIDAHGKIDVTKAPNFRFSPYSNQSHWIQGSNSQFGSAHSHALDLKSAFKPLPNLPGLSGLHSAFLPGYPTLSSANPLTSHFTPGQYSSANLGLPFPHGTYSGLPGYTGAMNGLIGTPFSLFPYRSSEARDLTKMAERDSTTDIEKK |
| Mme|XP_045157053.1 | MVEKIDLLKQGHKRYCRWRDCLCAKCMLIAERQRVMAAQVALRRQQAQEENEAKDLGMLYGPGGLLQVNSNNFSETSIGSNKRRNDQPDKATAMKQAKSDREHEDVERKTAAADKNSELTVSSTESDANKISISPRNAREVALQYPQTESQDADKSTDKKIQIICKLFPMLSVSSVNDILNSCDRDISKSIDFILSKNLNDSCSSVAMISSAMTFHPGIYGNAMHPSIYGSNAAFGRLRTFPACPIGGFRLPMTTVYNQIPSSSHVPYSIIGMSGINYADRSKPGYKAGETNTADGESKE |
| Oe|XP_048763391.1 | MSSDEEKGDSHSSVFMRASDRYPRTPKCARCRNHGVVSALKGHKRYCRWRDCVCAKCTLIAERQRVMAAQVALRRQQAQEENEARELGMLYGPSGLLQLNPETINLFPNAKKIIDTDKDEDDGPAVKRQKLDSTRTDSPVSRCSSEDNNERTYSPVESTGSPAPSPKFTDPPSPSEDKSEPFPKSFEEGLLAGSSKKNPIDMLQRIFPHMKKSVLQLILQGCNGDIVQTIEQVLSNHGSDQSSAASTSSSSFMPHPGLVSTMTNSSFRSAFSPISTLANAHTLNSMRYAWGGMGGRGLLAMPYPPVLPGLTLGAAYSNYNSLNSSSSGPKPFHYAMCPCCTTKPFSSSNSEKSSCIAE |
| Oe|XP_048761857.1 | MNPVTALYPVTEKGTRKPKCARCRNHGMVSWLKGHKRHCKFKDCNCAKCSLIAERQRVMAAQVALKRQQAAEDAIALGLRCATEGSIPIMTQGPLWGPGTVSPPEDKDSKGQDSETDDPDGNLSEDNEPTQNGISIAPEVTKPRHDTSSPDSTEENDSVKVKATAPTSYAHHLLPPTAFLPGRLSNLEILERIFPFHRKSVLELVLQGCNGDLVKAIEQFLSSQETMLSNQNVPHIKSDFRSHPYLQSFQYGTAMKSLNGLTRLSQSGPGSAFTPFSSAGPFNPGIHSAFQSHLSNFTADTLRPSYISGTARPDIFPPNSQLTYPRLHPFTSAPLPGFFSNSFPLTPYRMDMADVNSHKPISEKHTVKTLNSENGHESEMWDEGSRDRDRE |
| Oe|XP_048736153.1 | MCDRKYIETSVRRCSRCRNHDILVELKGHKNFCQYQNCSCQACALLDKKKEICRQAIALRRKQDKGKKGSSHDVQPASLRCYIRQHPYRNFPGHRISPPLEEPQVTTTLPSLQPAPNYLQTMQAKTPYNSHLRRYMNTTRNLVSGYPGIISPSVGMTMGTNTAPSFDPLMCTVYQPSHPGLRQPPYVQPRPCLCEPTYTTLKPVSHVGLHPSGSAPSFNHILSMPSWIENPLWYGQEQVPYRPWMSSQYIAPPANESQNRRM |
| Oe|XP_048736174.1 | MCDRKYIETSVRRCSRCRNHDILVELKGHKNFCQYQNCSCQACALLDKKKEICRQAIALRRKQDKGKKGSSHDVQPASLRCYIRQHPYRNFPGHRISPPLEEPQVTTTLPSLQPAPNYLQTMQAKTPYNSHLRRYMNTTRNLVSGYPGIISPSVGMTMGTNTAPSFDPLMCTVYQPSHPGLRQPPYVQPRPCLCEPTYTTLKPVSHVGLHPSGSAPSFNHILSMPSWIENPLWYGQEQVPYRPWMSSQYIAPPANESQNRRM |
| Sg|Sgl006992-mRNA1 | MSSDEEKGDSHSFMRASDRYPRTPKCARCRNHGVVSALKGHKRYCRWRDCVCAKCTLIAERQRVMAAQVALRRQQAQEENEARELGMLYGPSGLLHLNPETMNMFPDAKKIVEKNKDEDEPAAKRQKLDSLRTESPVSRCSSEDVNERTHSPVESTGSPAPSPKIPDSPTPNEDKSESFSKSPYEEGLLPGGSKKNPIDMLQRIFPHMKRSVLQLILQGCNGDVVQTIEQVLSNHGTDQSATSSSSSSSSFLPHPGLVSTMTNSSFRSAFSPISTLANAHTLNSMRYAWGGMGGRGLLAMPYPPVLPGLTLGAAYSNYSGLNSSSNGSKPFHYAMCPCCTTKPFSSSNSEKSSCIAE |
| Sg|Sgl014397-mRNA1 | MNPVTALYPITEKGTRKPKCARCRNHGMVSWLKGHKRHCKFKDCNCAKCSLIAERQRVMAAQVALKRQQAAEDAIALGLRCATEGSLPIMTQGPLWGPGTVSPPKEKDSKSQESETDEPEDNSSDENEPAQNGRLIAKEVTKPRHDTSSPDSTEENESVKVKVTAPTSYSVHSLLPPNAFLPGRLSNLEILERIFPFHRKSVLELVLQGCNGDLVKSIEQFLSSQETMISNQTLPHVKSDFRPHPYFQYGSAMKSLNGVTRLSPTGSASAFTPFSSAGPFTPGIHSAFQSHLTNFTADSLRPSYLQSVSGSVRPDIFPPNNQLTYPRLHPLTSAPLPGFFSGSFPLTPYRIDMGDSCRKTLSEKHSGKTVNTENGHESEMWDEGSRDRDRE |
| Sg|Sgl011295-mRNA1 | MDDKNIETEAEGSVRSVTVRRCNRCRNHGYLVELKGHKHFCQYRDCTCQACVVLEKRKEVCRQAIALRRKQDMAKNANGPSLPVLQLHSSRQHPYLRPLNPRDRQPFKEPHHISVTLPNLTPCPNYVKTMQIKTTYNSQMRNYTNITKNVVSQTMGMTTGAHGAPATEMKTLKAQTRMSICTPSQPATPGLHRLPNAQPIPCLCEPTYTTLRPVSHVGLYPSGSTSFNPSFSRATWMESPFWYGQEHGYFRQMMPITQWATASPANSSQSPGK |
| Cs|evm.model.Hic_asm_11.338 | MSSSEDERGQTDGGADGGTGDGVFMRSASDRYPRTPKCARCRNHGVVSALKGHKRYCRWRDCMCAKCTLIAERQRVMAAQVALRRQQAQEENEARDMGVLYGPNGLLQINPECASLFPDATKPLDTSKSRNDVDPTPERTTEDLPAAKRQKTESRKEESDRLSASSDDSRSVSPSYSDDKRLHDNGRSSPLRHSPSAYDDLTTFYDRNGRLSENWVLNLAQNSSKKQQHPLDMLCRIFPHKKRNVLELILQGCGGDTVQAIEQVLTTQREEEKTATSLMYPSNPSYPGLPNPLHNSVFKSAFSPISTLSAANTLSTMRYAWGGAAGRGLAMTMPYSHLIPGLSMGPSFGYGAVGTSGDKLSPYSMYPFWTAKPFSAKDGDKSSGCVSD |
| Cs|evm.model.Hic_asm_11.400 | MSSSEDERGQTDGGADGGTGDGVFMRSASDRYPRTPKCARCRNHGVVSALKGHKRYCRWRDCMCAKCTLIAERQRVMAAQVALRRQQAQEENEARDMGVLYGPNGLLQINPECASLFPDATKPLDTSKSRNDVDPTPERTTEDLPAAKRQKTESRKEESDRLSASSDDSRSVSPSYSDDKRLHDNGRSSPLRHSPSAYDDLTTFYDRNGRLSENWVLNLAQNSSKKQQHPLDMLCRIFPHKKRNVLELILQGCGGDTVQAIEQVLTTQREEEKTATSLMYPSNPSYPGLPNPLHNSVFKSAFSPISTLSAANTLSTMRYAWGGAAGRGLAMTMPYSHLIPGLSMGPSFGYGAVGTSGDKLSPYSMYPFWTAKPFSAKDGDKSSGCVSD |
| Cs|evm.model.Hic_asm_11.1174 | MNSLSHLYPVTEKGTRKPKCARCRNHGMVSWLKGHKRHCDFKDCTCAKCNLIAERQRVMAAQVALKRQQATEDAIALGIRACAGTDSSLPIMTEGPLWGPGTVSIPASQAEREHQERLSRQQSSPDSRSEDDDDISVCSEEGDSNLTKPEDGHRRIPTPSTPEKHDGKFDDRDTIIRSNVLKPAAFTPGRLTNLEILERVFPLHRKSVLELVLQGCNGDLVKAIEQFLSAQDTIDAHGKMDSSKAPSVRFNPYSNPPHWIQGSNPQISAHSHAFDLKSAFKPLPNFPALSGLHSAFLPGYPTLSSANPLTSQFTPGQYSTANLGLPFPHGTYTGLPGYTGTMGGLLGSPFSLLPYRNAEARDLTKIAERDSATEVEKK |
| Ap|evm.model.scaffold_95.77 | MSSDDEKSDGANGMFMRAADRYPRTPKCARCRNHGVVSALKGHKRYCRWRDCVCAKCTLIAERQRVMAAQVALRRQQAQEENEARELGMLYGNTGLLQINPETVNMFPEAKQYLESGKRETSDEYPASKKQKLDDHRDDLQPEATRPESRSPTPDVMDRPRSPADSAGSPEPSPRSCDPPSPRSPASKSDSKSPFDESTLAHYGGNKRQPIEMLARIFPHMKRSVLQLILQGCGGDVVQCIEQILNSHGEASGMSSAFAGSPLITPHGISNPLGAPSLKSAFSPIASSIANAHSLNSIRNAWGSMSRGLLAMPYPPAFPGLTLGSNYSAYSGLTSDSSSKPFPYSMYPYCPTKPFTASTSDKSGCLGE |
| Ap|evm.model.scaffold_95.76 | MNPVTLYPLTEKGSRKPKCARCRNHGMVSWLKGHKRHCKFKDCACQKCNLIAERQRIMAAQVALKRQQAAEDAIALGLRACAQDHINPVMTSGPLWGPGTVSPPQGDKEGRCDEEETNENTPKITEDSSEDDRVEASSPESVNEMTETPERPKPYSPYTSLTRPRDLPSATSFLPGHLNNLEILERVFPFQRKSVLELVLQGCNGDLVKAIEQFLSTQDTLMTQQTTPRLKQDFRSHPYFGQLPLHTLKSLNSKLPNGSSMRSAFTPFSSPAPMAHTGLHSAFTSPVNTMSCDALRAQMFPSGMRSSDVLPPSSQFPYPSFSHLTSGPLPGFMSSPFSLYPYRSGITDMNCFRKTSEKASERAALQESSQNVENWDESAKERDVE |
| Ap|evm.model.scaffold_235.403 | MSSKKETKPAVGLSPLRSPKCCRCRNHGIITVLKGHKRFCQWKNCTCDNCLLLMKRQQNSKEQIALRRLWKQEEDMGLVAPTPVSTDSLQILIHRYPHYNVDKLGAILKSCDGDTQKAIERIDAVTNRALDAITTEPKSGMTPSCAGDMCVSPGNTAGMGFVGFQGYNSGFHTPLPPPPSGVNPRASMQMYPRTPYMYPYSPPRFYPSPQQDIPYNGDFHGPAPRSGHSAQNFQTSRNAFYSECQSSVESLIGYPKASEAGVYQSRPSTASSDTEGNLVIDMKDEEESVCAEGLLTSYPTQ |
